# Pharmacological Interventions for Excessive Daytime Sleepiness in Adults with Narcolepsy: A Systematic Review and Network Meta-Analysis

**DOI:** 10.3390/jcm11216302

**Published:** 2022-10-26

**Authors:** Po-Yu Chien, Chan-Yen Kuo, Meng-Hsuan Lin, Yao-Jen Chang, Chin-Chuan Hung

**Affiliations:** 1 Department of Pharmacy, China Medical University, No. 100, Sec. 1, Jingmao Rd., Beitun Dist., Taichung 406040, Taiwan; 2 Department of Pharmacy, China Medical University Hospital, No. 2, Yude Rd., North Dist., Taichung 404332, Taiwan; 3 Department of Research, Taipei Tzu Chi Hospital, Buddhist Tzu Chi Medical Foundation, New Taipei City 231016, Taiwan; 4 School of Medicine, Buddhist Tzu Chi University, Hualien 97004, Taiwan; 5 Department of Surgery, Taipei Tzu Chi Hospital, Buddhist Tzu Chi Medical Foundation, New Taipei City 231016, Taiwan; 6 Department of Healthcare Administration, Asia University, 500, Lioufeng Rd., Wufeng, Taichung 41354, Taiwan

**Keywords:** narcolepsy, excessive daytime sleepiness, systematic review, network meta-analysis, cataplexy

## Abstract

Narcolepsy is a neurological disease characterized by a core symptom of excessive daytime sleepiness (EDS). Although effective pharmacological interventions for narcolepsy have been developed, a lack of comparative evidence supporting the relative efficacy among these medications leads to clinical treatment challenge. Therefore, we performed a network meta-analysis to overcome this lack of head-to-head comparisons. Databases were searched systematically for randomized controlled trials that compared pharmacological interventions for narcolepsy. The primary outcomes were changes in the Epworth Sleepiness Scale (ESS) and the Maintenance of Wakefulness Test (MWT). A random-effects frequentist network meta-analysis was conducted. A total of 19 RCTs involving 2504 patients were included. Solriamfetol achieved the highest ranking based on the P-scores, and was superior to pitolisant (MD −2.88, 95% CI −4.89–−0.88) and sodium oxybate (MD −2.56, 95% CI −4.62–−0.51) for ESS change. Consistently, solriamfetol achieved the highest ranking according to MWT change, and was superior to pitolisant (SMD 0.45, 95% CI 0.02–0.88) and modafinil (SMD 0.42, 95% CI 0.05–0.79). Although solriamfetol demonstrated superior efficacy in EDS improvement, evidence from the clustered ranking plot supported that efficacy–safety profiles of pitolisant, sodium oxybate, and modafinil are more balanced than solriamfetol. Therefore, the choice of medication for EDS in narcolepsy should be made on an individual basis.

## 1. Introduction

Narcolepsy is a neurological disease with a ubiquitous clinical feature of excessive daytime sleepiness (EDS). It is the second leading cause of disabling daytime sleepiness after obstructive sleep apnea [1,2]. Dysfunction of rapid eye movement (REM) in narcolepsy results in cataplexy, sleep paralysis, and hypnagogic hallucinations. According to the third edition of International Classification of Sleep Disorders (ICSD-3), narcolepsy type 1 is characterized by either hypocretin-1 deficiency in the cerebrospinal fluid or by cataplexy, which leads to a sudden loss of muscle tone with intact consciousness [3]. Conversely, narcolepsy type 2 is characterized by an absence of cataplexy, and normal hypocretin-1 levels, if measured.

Because the impacts of narcolepsy are debilitating and disrupt the social and occupational functioning of nearly half of patients with the condition, managing symptoms of narcolepsy, especially EDS, is of great clinical importance [4]. Nonpharmacological approaches, including regular napping and sleep hygiene improvement, can help with the symptoms of narcolepsy [5]. Nevertheless, pharmacological interventions remain a cornerstone in the treatment of narcolepsy.

Currently, multiple pharmacological interventions have been approved for the treatment of narcolepsy [4]. Modafinil, armodafinil, and solriamfetol are effective in controlling EDS associated with narcolepsy [6,7,8], whereas sodium oxybate, pitolisant, and lower-sodium oxybate play a role in treating EDS and REM-associated deficits, such as cataplexy [9,10,11]. Although multiple treatment options are available for patients with symptoms of narcolepsy, a lack of comparative evidence has yet to provide their relative efficacy and safety. This lack of head-to-head comparisons makes selecting the most appropriate treatment for each patient’s therapeutic goals difficult for patients, caretakers, and clinicians.

Previous systematic reviews investigating the effects of interventions on EDS have mainly focused on single intervention that compares to placebo or to active drugs [12,13]. In addition, new medications have been approved for the treatment of narcolepsy, and a previous study that compared multiple interventions in narcolepsy failed to account for the current therapeutic options [14]. Given the various interventions with unclear positions in relative ranking to choose from, we considered a network meta-analysis would fill this important evidence gap. A network meta-analysis is able to converge direct and indirect evidence, and further ranks the relative effects of multiple interventions [15]. Hence, we aimed to utilize a network meta-analysis approach to compare the efficacy and tolerability between pharmacological interventions for the treatment of narcolepsy.

## 2. Materials and Methods

### 2.1. Search Strategy

This network meta-analysis was conducted in accordance with the Preferred Reporting Items for Systematic Reviews and Meta-analysis guidelines (PRISMA) [16] and registered prospectively in PROSPERO (CRD42021273182). We searched electronic databases, namely PubMed, Web of Science, Embase, the Cochrane Central Register of Controlled Trials (CENTRAL), the China National Knowledge Infrastructure, the Psychology and Behavioral Sciences Collection, and Clinical Trials.gov, from inception to 1 January 2022. The key words we used to search the databases are listed in Supplementary Table S1.

All the studies identified through the electronic database search were compiled in the reference management tool, and duplicates were removed. The studies were subsequently screened at the title and abstract level. Finally, the full texts of the studies were thoroughly screened according to the prespecified eligibility criteria.

### 2.2. Eligibility Criteria

Randomized controlled trials of pharmacological interventions used for narcolepsy treatment in human patients were included. The included interventions comprising any medications approved for the treatment of narcolepsy; the comparison could be either placebo or active medication. The study population included adult patients (≥16 years of age) with narcolepsy, regardless of the presence of cataplexy. We accepted the definitions of narcolepsy used in the original studies, which generally corresponded to the current ICSD-3 criteria for narcolepsy type 1 or 2 [3]. Our two primary outcomes were subjective and objective daytime sleepiness, which were quantified as changes in the Epworth Sleepiness Scale (ESS) and Maintenance of Wakefulness Test (MWT; 20- or 40-min version) scores, respectively. The secondary outcomes were the change in cataplexy rate, Clinical Global Impression of Change (CGI-C), and adverse events (AEs). No limitations on language or publication year were applied. Given the elimination half-life of interventions and characteristics of narcolepsy, we assessed that carry-over effect and period effect were limited [17]. Therefore, trials employing a crossover design were included. Nevertheless, sensitivity tests were conducted to exclude crossover trials. When certain data were omitted or required further elucidation, we contacted the corresponding author of the study from which they came.

### 2.3. Study Quality and Risk of Bias

Risk-of-bias evaluation of the included studies was conducted using the Cochrane Risk of Bias 2 tool [18]. The following domains were evaluated: (1) appropriateness of randomization process, (2) participants’ and caretakers’ awareness of their assignment and deviations from the intervention, (3) availability of outcome data for all randomized participants and whether missingness depended on true value, (4) appropriateness of the method for measuring the outcome and the assessors’ awareness of the assignment, and (5) use of a prespecified outcome measure. The risk of bias in each domain was categorized as low risk, some concerns, or high risk. Trials considered to have a low risk of overall bias were required to have a low risk of bias for all domains. Trials that had some concerns in at least one domain, but no domains with a high risk of bias, were considered to have some concerns overall. Any study considered to have a high overall risk of bias had at least one domain graded as high risk. The risk-of-bias results are presented as plots by using the risk-of-bias visualization (robvis) web app [19]. A funnel plot and Egger’s test were applied to evaluate the risk of small-study effects when an individual meta-analysis included 10 or more studies [20]. A *p* value of ≥0.05 in Egger’s test indicated a low risk of small-study effects.

### 2.4. Outcomes and Data Extraction

Our primary objective was to investigate the relative efficacy of included interventions in alleviating EDS. The primary outcomes were changes in ESS and MWT from baseline to endpoint [21,22]. The secondary outcomes were change in cataplexy rate from baseline to endpoint, CGI-C, and AEs. The change in cataplexy rate was calculated as the changes in either daily or weekly numbers of cataplexy attacks. Supplementary Table S2 summarizes the categorization of AEs. We used the definitions of serious AEs that were used in the original trials.

Pairs of both bilingual English- and Chinese-speaking reviewers completed the literature screening, study appraisal, and data extraction independently. Discrepancies were resolved through mutual discussion, followed by confirmation by a third senior reviewer.

### 2.5. Statistical Analysis

A random-effects frequentist network meta-analysis was conducted to converge direct (within-trial comparisons) and indirect (between-trial comparisons) evidence [23]. The frequentist model generates P-scores, which can be used to rank interventions according to the extent of estimates. This approach is practical when direct comparisons are scarce from clinical trials. For changes in ESS, MWT, cataplexy rate, and CGI-C, a higher P-score indicated greater alleviation of EDS or cataplexy. For AEs, a higher P-score indicated a lower incidence of AEs. League tables were constructed based on the P-scores; a higher position indicated a higher P-score.

Medians were converted to means and standard deviations [24]. For continuous outcomes, ESS is presented as mean difference (MD) with 95% confidence intervals (CIs); MWT and cataplexy rate are presented as standardized mean difference (SMD) due to the combination of 20- and 40-min versions, as well as of daily and weekly attack numbers, respectively. For binary outcomes, risk ratio (RR) was used. Local inconsistency was measured using the back-calculation approach [25]; global inconsistency was assessed using the design-by-treatment model, and visualized as the net heat matrix [26]. A *p* value of ≤0.05 indicated a significant inconsistency. We utilized R (version 4.0.3) and the netmeta package (version 2.0-1) for all the analyses [27].

## 3. Results

### 3.1. Study Selection and Baseline Characteristics

**Figure 1:**
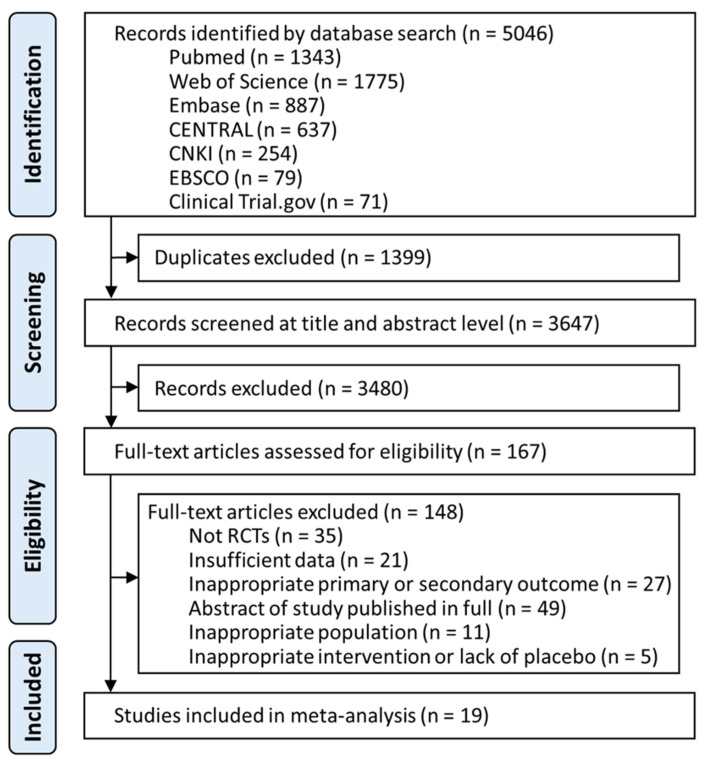
Summary of study selection. Figure demonstrates the flow of retrieval and identification of included trials. Overall, six pharmacological interventions were fitted into the analyses, including armodafinil, lower-sodium oxybate, modafinil, pitolisant, sodium oxybate, and solriamfetol. (Listed in alphabetical order). CENTRAL, Cochrane Central Register of Controlled trials; CNKI, China National Knowledge Infrastructure; EBSCO, Psychology and Behavioral Sciences Collection.

A total of 5046 studies were identified from the databases. After removing duplicates and screening titles and abstracts, 167 articles were assessed for eligibility. Further full text assessment excluded 148 articles (Supplementary Table S3). Ultimately, 19 studies, with a total of 2504 participants that met eligibility criteria, were included in the network meta-analysis (Figure 1, Supplementary Table S4).

Overall, the mean or median ages in the studies ranged from 33.0 to 49.1 years, and the genders of participants did not differ significantly among the studies (*p* = 0.295). The trials were primarily conducted in North America (46.4%) and Europe (42.8%). The quality assessment revealed that five included trials showed a high risk of bias. The domain wherein the studies exhibited a high risk of bias included deviations from intended interventions, which were attributed to the nature of the crossover designs (Supplementary Figure S1).

### 3.2. Primary Outcomes

#### 3.2.1. Change in Epworth Sleepiness Scale

Five pharmacological interventions studied in 13 of the included trials contributed to the network (N = 1818, Figure 2a, Supplementary Table S4) [28,29,30,31,32,33,34,35,36,37,38,39,40]. Compared to placebo, solriamfetol (MD −4.76, 95% CI −6.11–−3.42), modafinil (MD −3.37, 95% CI −4.42–−2.33), lower-sodium oxybate (MD −3.00 95% CI −5.11–−0.89), sodium oxybate (MD −2.20 95% CI −3.75–−0.65), and pitolisant (MD −1.88, 95% CI −3.36–−0.40) showed superiority in ESS improvement (Figure 3a). Solriamfetol achieved the highest ranking based on the P-scores of the interventions, suggesting that among the interventions, it was the most effective in reducing ESS (Table 1). Moreover, solriamfetol was superior to sodium oxybate (MD −2.56, 95% CI −4.62–−0.51) and pitolisant (MD −2.88, 95% CI −4.89–−0.88) for the reduction of ESS. The global Q score for inconsistency was 3.88 (*p* = 0.1434), and hotspots of inconsistency were minimal from the net heat plot (Supplementary Figure S2). Back-calculation and Egger’s test revealed absence of local inconsistency (Supplementary Table S5) and small-study effects (*p* = 0.7886, Supplementary Figure S3), respectively.

Subgroup analyses were undertaken to further evaluate our findings. When the analyses were restricted to trials that recruited participants with cataplexy, three interventions studied in four trials connected to the evidence network (Supplementary Figure S4, Supplementary Table S6) [28,34,39,40]. Pitolisant (MD –3.50, 95% CI −5.76–−1.24), lower-sodium oxybate (MD −3.00, 95% CI −4.70–−1.30), and sodium oxybate (MD −2.22, 95% CI −3.49–−0.96) resulted in significantly greater improvement in ESS than did placebo (Supplementary Figure S5, Supplementary Table S7).

The trends in estimated treatment effects remained consistent in the sensitivity analyses, excluding trials with crossover designs (Supplementary Figure S6, Supplementary Table S8) and withdrawal designs (Supplementary Figure S7, Supplementary Table S9).

#### 3.2.2. Change in Maintenance of Wakefulness Test

Five pharmacological interventions studied in 12 trials contributed to the network (N = 1931, Figure 2b, Supplementary Table S4) [29,30,31,32,33,34,35,36,37,39,41,42]. As compared to placebo, solriamfetol (SMD 0.97, 95% CI 0.67–1.28), armodafinil (SMD 0.58, 95% CI 0.12–1.05), modafinil (SMD 0.55, 95% CI 0.34–0.77), sodium oxybate (SMD 0.55, 95% CI 0.23–0.87), and pitolisant (SMD 0.52, 95% CI 0.22–0.83) showed superiority in MWT improvement (Figure 3b). Among the interventions, solriamfetol achieved the highest ranking for maintaining wakefulness (Table 1), and was superior to modafinil (SMD 0.42, 95% CI 0.05–0.79) and pitolisant (SMD 0.45, 95% CI 0.02–0.88). The global Q score for inconsistency was 1.93 (*p* = 0.7489), and hotspots of inconsistency were minimal from the net heat plot (Supplementary Figure S8). No evidence of network inconsistency (Supplementary Table S10), nor small-study effects (*p* = 0.6015, Supplementary Figure S9), was uncovered.

When the analyses were restricted to the trials that recruited participants with cataplexy, two trials with two interventions connected to the evidence network (Supplementary Figure S10, Supplementary Table S11) [34,39]. Patients treated with pitolisant or sodium oxybate showed significantly better MWT than did those who received placebo (Supplementary Figure S11). In addition, pitolisant was superior to sodium oxybate in maintaining wakefulness (Supplementary Table S12).

Sensitivity analyses that excluded trials with crossover and withdrawal designs showed a consistent trend of estimated treatment effects (Supplementary Figures S12 and S13); yet, compared to modafinil (SMD 0.29, 95% CI −0.09– 0.68) and pitolisant (SMD 0.33, 95% CI −0.11– 0.77), solriamfetol did not reach significance in MWT improvement when excluding crossover-designed trials (Supplementary Table S13). The difference between solriamfetol and modafinil approached, but did not reach, significance (SMD 0.40, 95% CI 0.00–0.80) in MWT improvement when excluding withdrawal-designed trials (Supplementary Table S14).

### 3.3. Secondary Outcomes

#### 3.3.1. Change in Cataplexy Rate

**Figure 2:**
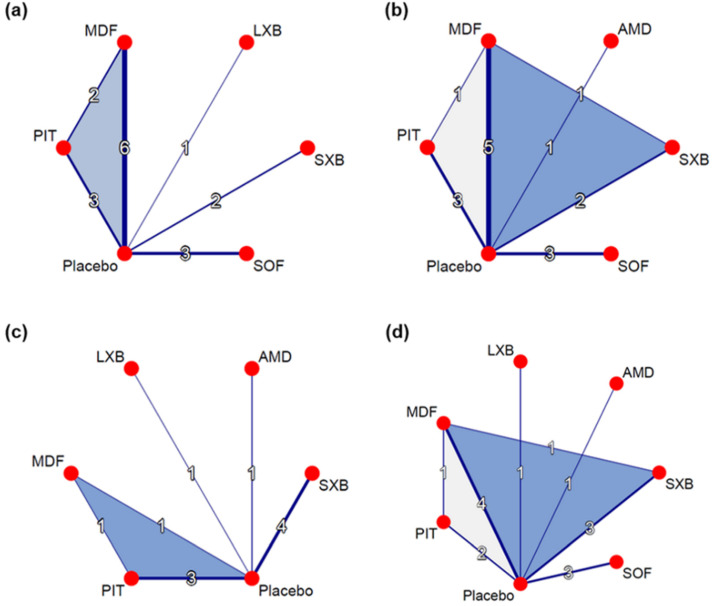
Network graphs for outcomes of interest. (**a**) Change in ESS. (**b**) Change in MWT. (**c**) Change in cataplexy rate. (**d**) CGI-C. Nodes indicate interventions, and lines connecting these nodes indicate head-to-head comparisons. The width of lines corresponds to the number of trials studying the comparison. ESS, Epworth Sleepiness Scale; MWT, Maintenance of Wakefulness Test; CGI-C, Clinical Global Impression of Change; MDF, modafinil; LXB, lower-sodium oxybate; SXB, sodium oxybate; SOF, solriamfetol; PIT, pitolisant; AMD, armodafinil.

Five pharmacological interventions studied in nine trials contributed to the network (N = 1082, Figure 2c, Supplementary Table S4) [28,32,33,34,40,42,43,44,45]. Pitolisant (SMD −0.72, 95% CI −1.13–−0.32) and sodium oxybate (SMD −0.40, 95% CI −0.76–−0.04) contributed significantly greater decreases in cataplexy rate than did placebo. The difference between lower-sodium oxybate and placebo approached, but did not reach, significance (SMD −0.64, 95% CI −1.27–0.00). Based on the P-scores of the interventions, pitolisant achieved the highest ranking for reducing cataplexy rate (Table 1); however, analyses combining direct and indirect evidence revealed no differences between pitolisant and each of the comparators. The global Q score for inconsistency was 1.19 (*p* = 0.2755). Although hotspots of inconsistency were unable to be assessed due to the insufficient number of designs available by the program specification, no evidence of network inconsistency was uncovered (Supplementary Table S15); the risk of small-study effects could not be evaluated because the number of trials was less than ten.

When analyses were restricted to studies of interventions that had been approved for the treatment of cataplexy, three interventions studied in eight trials connected to the evidence network (Supplementary Figure S14, Supplementary Table S16) [28,32,33,34,40,43,44,45]. The patients treated with pitolisant, lower-sodium oxybate, and sodium oxybate had significantly lower rates of cataplexy than did those who received placebo (Supplementary Figure S15); pitolisant maintained the highest ranking (Supplementary Table S17).

The trends in estimated treatment effects remained consistent in the sensitivity analyses, excluding trials with crossover designs (Supplementary Figure S16, Supplementary Table S18) and withdrawal designs (Supplementary Figure S17, Supplementary Table S19).

#### 3.3.2. Clinical Global Impression of Change

**Figure 3:**
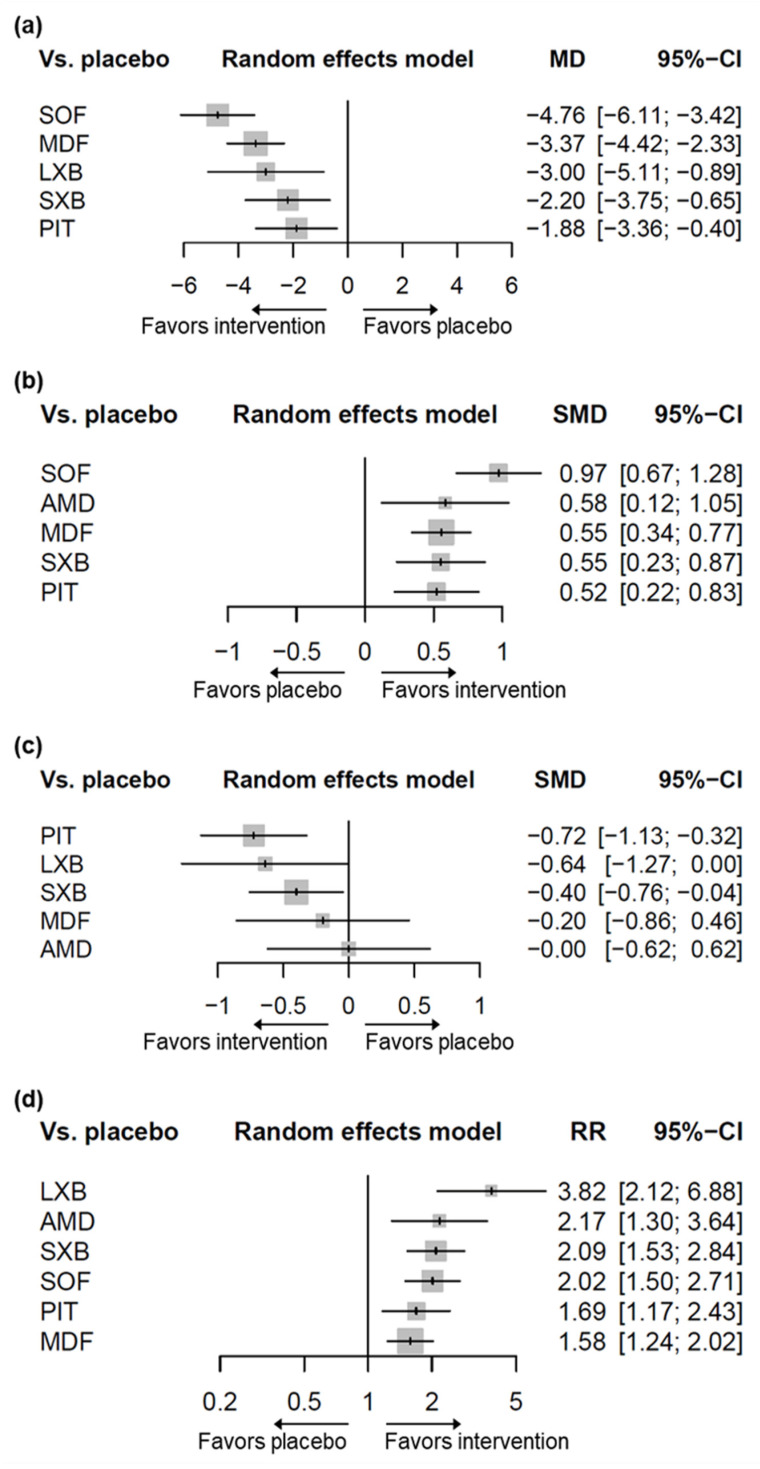
Forest plots for the outcomes of interventions compared to placebo. (**a**) Change in ESS. (**b**) Change in MWT. (**c**) Change in cataplexy rate. (**d**) CGI-C. ESS, Epworth Sleepiness Scale; MWT, Maintenance of Wakefulness Test; CGI-C, Clinical Global Impression of Change; SOF, solriamfetol; LXB, lower-sodium oxybate; MDF, modafinil; SXB, sodium oxybate; PIT, pitolisant; AMD, armodafinil; MD, mean difference; SMD, standardized mean difference; RR, risk ratio; CI, confidence interval.

Six pharmacological interventions studied in 12 trials contributed to the network (N = 1973, Figure 2d, Supplementary Table S4) [28,29,30,31,32,34,35,36,39,40,41,42]. All the interventions (lower-sodium oxybate [RR 3.82, 95% CI 2.12–6.88], armodafinil [RR 2.17, 95% CI 1.30–3.64], sodium oxybate [RR 2.09, 95% CI 1.53–2.84], solriamfetol [RR 2.02, 95% CI 1.50–2.71], pitolisant [RR 1.69, 95% CI 1.17–2.43], and modafinil [RR 1.58, 95% CI 1.24–2.02]) resulted in significantly greater improvement of CGI-C than did placebo (Figure 3d). Lower-sodium oxybate achieved the highest ranking for the improvement of CGI-C (Table 1), and was superior to pitolisant (RR 2.27, 95% CI 1.14–4.52) and modafinil (RR 2.42, 95% CI 1.28–4.57). The global Q score for inconsistency was 12.93 (*p* = 0.0116). Hotspots of inconsistency existed from the heat plot (Supplementary Figure S18); significance was identified in the comparison of modafinil with sodium oxybate (*p* = 0.0259), and of sodium oxybate with placebo (*p* = 0.0299, Supplementary Table S20). No evidence of small-study effects was uncovered (*p* = 0.5161, Supplementary Figure S19).

When the analyses were restricted to the trials that recruited participants with the presence of cataplexy, three interventions studied in four trials connected to the network (Supplementary Figure S20, Supplementary Table S21) [28,34,39,40]. Lower-sodium oxybate, pitolisant, and sodium oxybate were superior to placebo in CGI-C improvement (Supplementary Figure S21); lower-sodium oxybate achieved the highest ranking for the improvement of CGI-C, and was superior to sodium oxybate (Supplementary Table S22).

The trends in estimated treatment effects remained consistent in the sensitivity analyses that excluded trials with crossover designs (Supplementary Figure S22, Supplementary Table S23). Excluding withdrawal-designed trials resulted in the exclusion of lower-sodium oxybate in the sensitivity analysis. Nevertheless, the estimated effects remained consistent among other interventions (Supplementary Figure S23, Supplementary Table S24).

To address the issue of inconsistency, we determined that excluding a minimum of three trials yielded a nonsignificant disagreement between the direct and indirect evidence (Supplementary Table S25) [36,39,40]. Reassuringly, the estimated treatment effects of all the interventions remained significant (Supplementary Figure S24); lower-sodium oxybate remained superior to pitolisant and modafinil for the improvement in CGI-C (Supplementary Table S26).

#### 3.3.3. Adverse Events

The patients treated with sodium oxybate, armodafinil, or solriamfetol exhibited a significantly higher rate of gastrointestinal AEs than did those who received placebo (Supplementary Figure S25); sodium oxybate was also associated with a higher incidence of gastrointestinal AEs than were modafinil and pitolisant (Supplementary Table S27). None of the interventions were associated with increases in the incidence of immunologic and musculoskeletal AEs relative to placebo (Supplementary Figures S26 and S27, Supplementary Tables S28 and S29). Solriamfetol was associated with a significantly higher incidence of neurological and psychological AEs than were modafinil and placebo (Supplementary Figures S28 and S29, Supplementary Tables S30 and S31), and a higher incidence of sleep-related AEs than was placebo (Supplementary Figure S30, Supplementary Table S32). Sodium oxybate was associated with a higher incidence of AEs in the “other” category than were placebo and modafinil (Supplementary Figure S31, Supplementary Table S33). Modafinil was associated with a higher incidence of serious AEs than was sodium oxybate (Supplementary Figure S32, Supplementary Table S34). Sodium oxybate was associated with significantly more AEs resulting in participant withdrawal from the trial than were placebo and lower-sodium oxybate (Supplementary Figure S33, Supplementary Table S35). Solriamfetol was associated with a higher rate of occurrence of any AE than was lower-sodium oxybate (Supplementary Figure S34, Supplementary Table S36).

### 3.4. Three-Dimensional Clustered Ranking Plot

**Figure 4:**
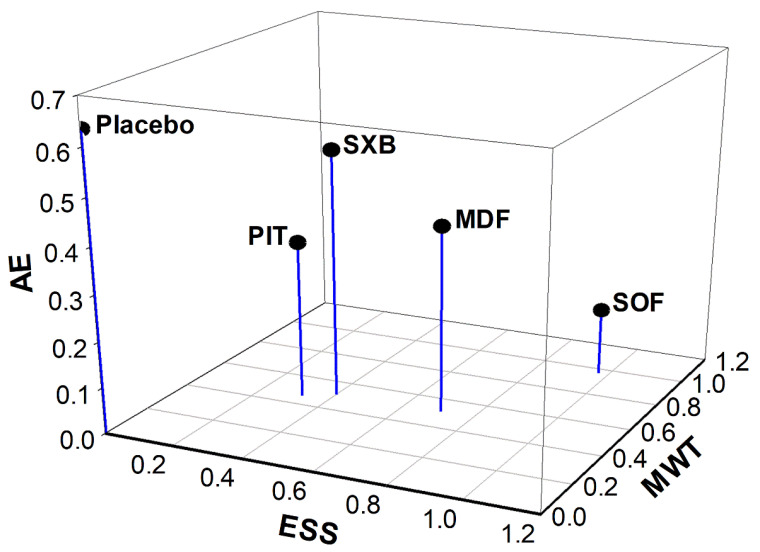
Three-dimensional clustered ranking plot converging P-scores of ESS change, MWT change, and AE for applicable interventions. ESS, Epworth Sleepiness Scale; MWT, Maintenance of Wakefulness Test; AE, adverse event; SOF, solriamfetol; MDF, modafinil; SXB, sodium oxybate; PIT, pitolisant. The X-, Y-, and Z-axis is ESS, MWT, and AE, respectively. Category of any AE that occurred to the participants was utilized to represent AE in the scatter plot. Length for interventions to origin of coordinates in planar (X-Y): SOF = 1.37; MDF = 0.87; SXB = 0.64; PIT = 0.56; placebo = 0.003. Length for interventions to origin of coordinates in 3D (X-Y-Z): SOF = 1.38; MDF = 0.96; SXB = 0.84; PIT = 0.66; placebo = 0.64.

A three-dimensional (3D) clustered ranking plot was constructed to compare the multiple outcomes of the interventions (Figure 4). Changes in ESS and MWT were plotted on the X-axis and Y-axis, respectively, to evaluate the efficacy of the interventions in alleviating EDS. The P-scores of solriamfetol, pitolisant, modafinil, and sodium oxybate were analyzed based on their reports in both primary outcomes. Compared to placebo, solriamfetol was plotted furthest from the origin of coordinates in the X–Y planar view, which suggests that among the interventions, it was the most effective in alleviating EDS. To give a comprehensive view regarding AEs, the occurrence of any AEs was utilized. Any AE was quantified as the number of participants who experienced at least one AE, and was plotted on the Z-axis. Regarding the safety of the interventions, solriamfetol showed the lowest P-score according to AEs; however, its overall distance from the origin remained the longest in the 3D perspective. The corresponding P-scores in AE for pitolisant, modafinil, and sodium oxybate reflected a probably lower incidence of AEs, suggesting that their efficacy–safety profiles were more balanced than that of solriamfetol.

## 4. Discussion

Given the substantial disease burden of narcolepsy, identifying the most effective medication for alleviating EDS is crucial. By using the network meta-analysis approach, we overcame the paucity of head-to-head comparisons of interventions. Direct and indirect comparisons were incorporated to rank the relative efficacy of the interventions for alleviating EDS. Our findings provide evidence that solriamfetol achieved the highest ranking among the comparators, and was superior to sodium oxybate and pitolisant according to the change in ESS, and to modafinil and pitolisant according to the change in MWT; the disagreements between direct and indirect evidence were nonsignificant, suggesting minimal inconsistency.

Different pharmacotherapeutic strategies have been applied to manage narcolepsy with and without cataplexy [46,47]. Previous meta-analyses have revealed that, for alleviating EDS in patients with narcolepsy, sodium oxybate and pitolisant are both superior to placebo [10,48]. However, these studies evaluated the pooled effects of the interventions regardless of the presence of cataplexy. Our meta-analysis was the first to account for the importance of cataplexy in the treatment of narcolepsy by subgrouping trials that recruited participants with narcolepsy and concurrent cataplexy. Intriguingly, we determined that patients with narcolepsy and cataplexy treated with pitolisant achieved significantly higher MWT than did those treated with sodium oxybate. Furthermore, regarding the change in ESS, pitolisant achieved the highest P-score, but did not show superiority over other comparators. These findings could support decision making for the pharmacological treatment of patients with cataplexy.

Our systematic review and network meta-analysis of randomized controlled trials involving patients with narcolepsy is the largest to date. We note our findings regarding the efficacy of pitolisant, modafinil, and sodium oxybate in alleviating EDS are coincided with another previous network meta-analysis [14], which reported that patients treated with pitolisant (up to 40 mg/d), modafinil (200–400 mg/d), or sodium oxybate (9 g/d) experienced significantly greater alleviation of EDS than did those who received placebo, but no differences were identified among the interventions. Nevertheless, the previous network meta-analysis only included studies published up to January 2017 and did not, therefore, include the recent trials examining solriamfetol and lower-sodium oxybate [28,29,30,31]. Our study included studies published since the publication of the previous network meta-analysis. Moreover, one additional randomized controlled trial comparing modafinil and placebo was included through our database search [38].

Occupational and social dysfunction can be detrimental in people with narcolepsy. Therefore, health-related quality of life (HRQoL) assessment plays a key role in evaluating the effectiveness of narcolepsy treatments. Emerging studies have incorporated HRQoL evaluations into their outcome measures [28,49]. However, the existing measurement tools for HRQoL are diverse, and the lack of specificity may be a concern in assessing the precise effects of narcolepsy on HRQoL [50]. Additionally, the change in ESS score and the mean sleep latency on MWT may not perfectly reflect the clinical meaningfulness of treatment effects for affected patients. Thus, developing a specific tool for detecting HRQoL impairment due to sleep-related dysfunction associated with narcolepsy is warranted. Also, patient-reported outcome measures are needed in future clinical trials.

Randomized withdrawal design (RWD) provides enrichment of apparent responders and, therefore, minimizes the sample size in clinical trials [51]. It is an established design for studies evaluating the efficacy of drugs used in the psychiatry and neurology areas. Notably, two trials with RWD, which studied lower-sodium oxybate and modafinil, were included in our analyses [28,37]. Given the lack of methods addressing issues regarding the incorporation of studies with traditional RCT design and RWD in the network meta-analysis, sensitivity tests were conducted to discover the possible impacts toward this incorporation. Reassuringly, results demonstrated that solriamfetol remained the top-ranking in both primary outcomes, and was superior to sodium oxybate and pitolisant in ESS change, as well as to pitolisant in MWT change.

The main strengths of our study are our search of a wide range of studies, and the inclusion of the latest approved interventions in a network meta-analysis. To enrich the findings, we did not set any restrictions on language. Furthermore, the use of network meta-analysis allowed us to explore the relative benefits of the interventions for narcolepsy. In addition, our study involved the first-ever construction of a 3D-clustered ranking plot designed to examine the relative efficacy or safety of the interventions in terms of the three outcomes of interest. According to our model, solriamfetol achieved the highest P-scores in combining ESS and MWT change. However, when the P-scores for AE were considered, the efficacy–safety profiles of pitolisant, sodium oxybate, and modafinil were more balanced than that of solriamfetol. Therefore, interventions for EDS should be selected according to individual clinical circumstances and the preferences of the patients and clinicians.

This study has some limitations. Firstly, randomized clinical trials for the treatment of narcolepsy recruited participants with concurrent cataplexy, or regardless of cataplexy status. Thus, we were unable to compare the efficacy of the interventions for alleviating EDS in patients with narcolepsy but absence of cataplexy in the subgroup analyses. Although EDS-specific interventions are frequently used for these patients [4], additional studies that employ more specific definitions of cataplexy status are warranted. Secondly, patients with narcolepsy commonly experience disturbed nocturnal sleep (DNS). Our outcomes of interest did not include change in DNS due to the lack of relevant documentation in the clinical trials. However, evidence supports that sodium oxybate is currently the drug of choice against DNS [4]. Thirdly, few of the trials compared the interventions with active controls. Consequently, the relative efficacy of each intervention was mainly analyzed through indirect comparisons. Although inconsistency in our primary outcomes was minimal, network meta-analyses should not be entirely substituted for head-to-head comparisons. Additional direct comparisons of pharmacological interventions for narcolepsy are warranted. Fourthly, despite the common use in clinical practice of amphetamine-type stimulants, they were not included in our analyses due to the lack of studies. Therefore, we are unable to evaluate the relative efficacy of amphetamines among other interventions for narcolepsy. Fifthly, due to the lack of individual participant data, intervening factors for the efficacy of interventions, including gender, age, and ethnicity, were not able to be addressed. As such, awareness should be taken when interpreting the findings of this study.

## 5. Conclusions

The results of our network meta-analysis suggest that for improvement in EDS, solriamfetol may be superior to pitolisant and sodium oxybate in ESS change, and to pitolisant and modafinil in MWT change. When AEs were considered, the efficacy–safety profiles of pitolisant, sodium oxybate, and modafinil were more balanced than that of solriamfetol. Treatment selection should be tailored on an individual basis and according to the therapeutic goals of the patients and clinicians.

## Figures and Tables

**Table 1:** League tables comparing estimated treatment effects combining direct and indirect evidence.

Change in ESS						
**Solriamfetol**						
−1.39 (−3.10–0.31)	**Modafinil**					
−1.76 (−4.27–0.74)	−0.37 (−2.73–1.99)	**Lower-sodium** **oxybate**				
**−2.56** **(−4.62–−0.51)**	−1.17 (−3.04–0.70)	−0.80 (−3.42–1.82)	**Sodium** **oxybate**			
**−2.88** **(−4.89–−0.88)**	−1.49 (−3.07–0.09)	−1.12 (−3.70–1.46)	−0.32 (−2.46–1.82)	**Pitolisant**		
**−4.76** **(−6.11–−3.42)**	**−3.37** **(−4.42–−2.33)**	**−3.00** **(−5.11–−0.89)**	**−2.20** **(−3.75–−0.65)**	**−1.88** **(−3.36–−0.40)**	**Placebo**	
**Change in MWT**						
**Solriamfetol**						
0.39 (−0.17–0.94)	**Armodafinil**					
**0.42** **(0.05–0.79)**	0.03 (−0.48–0.54)	**Modafinil**				
0.42 (−0.02–0.87)	0.03 (−0.53–0.59)	0.00 (−0.35–0.35)	**Sodium** **oxybate**			
**0.45** **(0.02–0.88)**	0.06 (−0.49–0.62)	0.03 (−0.32–0.38)	0.03 (−0.41–0.47)	**Pitolisant**		
**0.97** **(0.67–1.28)**	**0.58** **(0.12–1.05)**	**0.55** **(0.34–0.77)**	**0.55** **(0.23–0.87)**	**0.52** **(0.22–0.83)**	**Placebo**	
**Change in cataplexy rate**						
**Pitolisant**						
−0.09 (−0.84–0.67)	**Lower-sodium** **oxybate**					
−0.32 (−0.86–0.21)	−0.24 (−0.97–0.49)	**Sodium** **oxybate**				
−0.53 (−1.19–0.13)	−0.44 (−1.35–0.48)	−0.20 (−0.95–0.55)	**Modafinil**			
−0.72 (−1.46–0.01)	−0.64 (−1.52–0.25)	−0.40 (−1.11–0.31)	−0.20 (−1.10–0.71)	**Armodafinil**		
**−0.72** **(−1.13–−0.32)**	−0.64 (−1.27–0.00)	**−0.40** **(−0.76–−0.04)**	−0.20 (−0.86–0.46)	−0.00 (−0.62–0.62)	**Placebo**	
**CGI-C**						
**Lower-sodium** **oxybate**						
1.76 (0.80–3.85)	**Armodafinil**					
1.83 (0.94–3.55)	1.04 (0.57–1.90)	**Sodium** **oxybate**				
1.90 (0.98–3.66)	1.08 (0.59–1.96)	1.04 (0.68–1.59)	**Solriamfetol**			
**2.27** **(1.14–4.52)**	1.29 (0.68–2.42)	1.24 (0.78–1.98)	1.19 (0.75–1.91)	**Pitolisant**		
**2.42** **(1.28–4.57)**	1.37 (0.78–2.43)	1.32 (0.91–1.91)	1.27 (0.87–1.87)	1.07 (0.74–1.54)	**Modafinil**	
**3.82** **(2.12–6.88)**	**2.17** **(1.30–3.64)**	**2.09** **(1.53–2.84)**	**2.02** **(1.50–2.71)**	**1.69** **(1.17–2.43)**	**1.58** **(1.24–2.02)**	**Placebo**

ESS, Epworth Sleepiness Scale; MWT, Maintenance of Wakefulness Test; CGI-C, Clinical Global Impression of Change. The league tables contain the network estimates from network meta-analysis in the lower triangle. Interventions are ranked in a descending order of P-scores for each network. Estimates for change in ESS are shown in mean difference with 95% confidence interval. Estimates for change in MWT and cataplexy rate are shown in standardized mean difference with 95% confidence interval. Estimates for CGI-C are shown in risk ratio with 95% confidence interval.

## Data Availability

The contributions presented in the study are included in the article and Supplementary Information. Further data that support the findings of this study are available from the corresponding author upon reasonable request.

## Supplementary Materials

The following supporting information can be downloaded at: https://www.mdpi.com/article/10.3390/jcm11216302/s1, Table S1: Literature search terms; Table S2: Categories of adverse events; Table S3: Reasons for exclusion; Table S4: Summary of trials and pharmacological interventions that included in the network meta-analysis; Figure S1: Cochrane risk of bias appraisal. ESS change—Figure S2: Net heat plot; Table S5: Assessment of inconsistency; Figure S3: Funnel plot; Figure S4: Network graph-Trials included participants with cataplexy; Table S6: Assessment of inconsistency-Trials included participants with cataplexy; Figure S5: Forest plot-Trials included participants with cataplexy; Table S7: League table:-Trials included participants with cataplexy; Figure S6: Forest plot-crossover design excluded; Table S8: League table-crossover design excluded; Figure S7: Forest plot-withdrawal design excluded; Table S9: League table-withdrawal design excluded. MWT change—Figure S8: Net heat plot; Table S10: Assessment of inconsistency; Figure S9: Funnel plot; Figure S10: Network graph-Trials included participants with cataplexy; Table S11: Assessment of inconsistency-Trials included participants with cataplexy; Figure S11: Forest plot-Trials included participants with cataplexy; Table S12: League table-Trials included participants with cataplexy; Figure S12: Forest plot-crossover design excluded; Table S13: League table-crossover design excluded; Figure S13: Forest plot-withdrawal design excluded; Table S14: League table-withdrawal design excluded. Cataplexy rate change—Table S15: Assessment of inconsistency; Figure S14: Network graph-FDA approved Interventions for cataplexy; Table S16: Assessment of inconsistency-FDA approved Interventions for cataplexy; Figure S15: Forest plot-FDA approved Interventions for cataplexy; Table S17: League table-FDA approved Interventions for cataplexy; Figure S16: Forest plot-crossover design excluded; Table S18: League table-crossover design excluded; Figure S17: Forest plot-withdrawal design excluded; Table S19: League table-withdrawal design excluded. CGI-C—Figure S18: Net heat plot; Table S20: Assessment of inconsistency; Figure S19: Funnel plot; Figure S20: Network graph-Trials included participants with cataplexy; Table S21: Assessment of inconsistency-Trials included participants with cataplexy; Figure S21: Forest plot-Trials included participants with cataplexy; Table S22: League table-Trials included participants with cataplexy; Figure S22: Forest plot-crossover design excluded; Table S23: League table-crossover design excluded; Figure S23: Forest plot-withdrawal design excluded; Table S24: League table-withdrawal design excluded; Table S25: Assessment of inconsistency-Cook 2002, Ahmed 2005a, and Gross 2000 excluded; Figure S24: Forest plot-Cook 2002, Ahmed 2005a, and Gross 2000 excluded; Table S26: League table-Cook 2002, Ahmed 2005a, and Gross 2000 excluded. Adverse events—Figure S25: Forest plot-gastrointestinal; Table S27: League table-gastrointestinal; Figure S26: Forest plot-immunologic; Table S28: League table-immunologic; Figure S27: Forest plot-musculoskeletal; Table S29: League table-musculoskeletal; Figure S28: Forest plot: neurological; Table S30: League table-neurological; Figure S29: Forest plot-psychological; Table S31: League table-psychological; Figure S30: Forest plot-sleep-related; Table S32: League table-sleep-related; Figure S31: Forest plot-other; Table S33: League table-other; Figure S32: Forest plot-serious; Table S34: League table-serious; Figure S33: Forest plot-trial withdrawal due to AE; Table S35: League table-trial withdrawal due to AE; Figure S34: Forest plot-any AE occurred; Table S36: League table-any AE occurred.
