# Supplementary material for: Pharmacological Interventions for Excessive Daytime Sleepiness in Adults with Narcolepsy: A Systematic Review and Network Meta-Analysis
Source: J Clin Med. 2022 Oct 26;11(21):6302. doi: 10.3390/jcm11216302 (PMC9654433; doi:10.3390/jcm11216302)
Supplement: Supplementary file 1 [file jcm-11-06302-s001.zip › jcm-1969779-supplementary.pdf]

Journal of Clinical Medicine

Supplementary materials

Article

Pharmacological Interventions for Excessive Daytime Sleepiness in Adults with Narcolepsy: A Systematic Review and Network Meta-Analysis

Po-Yu Chien [1,2,†], Chan-Yen Kuo [3,†], Meng-Hsuan Lin [1], Yao-Jen Chang [4,5] and Chin-Chuan Hung [1,2,6,*]

[1] Department of Pharmacy, China Medical University, No. 100, Sec. 1, Jingmao Rd., Beitun Dist., Taichung 406040, Taiwan
[2] Department of Pharmacy, China Medical University Hospital, No. 2, Yude Rd., North Dist., Taichung 404332, Taiwan
[3] Department of Research, Taipei Tzu Chi Hospital, Buddhist Tzu Chi Medical Foundation, New Taipei City 231016, Taiwan
[4] School of Medicine, Buddhist Tzu Chi University, Hualien 97004, Taiwan
[5] Department of Surgery, Taipei Tzu Chi Hospital, Buddhist Tzu Chi Medical Foundation, New Taipei City 231016, Taiwan
[6] Department of Healthcare Administration, Asia University, 500, Lioufeng Rd., Wufeng, Taichung 41354, Taiwan
* Correspondence: cc0206hung@gmail.com
† These authors contributed equally to this study.

Contents

Literature search terms……………………..……………..…………..…………….3
Categories of adverse events…………………….………………………….…..………5
Reasons for exclusion……………………….……………………………………………6
Summary of trials that included in the network meta-analysis…………..………10
Cochrane risk of bias appraisal……..………...………………………………………..15
Change in Epworth Sleepiness Scale 16
Change in Maintenance of Wakefulness Test... 27
Change in cataplexy rate.. 38
Clinical Global Impression of Change 47
Adverse events 61
References.. 81

Supplementary Table S1 – **Literature search terms**

Database	Search terms	Result
PubMed	(solriamfetol OR JZP-110 OR ADX-N05 OR Sunosi) OR (modafinil OR provigil) OR (armodafinil OR nuvigil) OR (pitolisant OR wakix) OR (methylphenidate OR “methylphenidate hydrochloride” OR ritalin OR “ritalin LA” OR concerta) OR (amphetamine OR “amphetamine sulfate” OR adzenys OR dyanavel OR evekeo) OR (dextroamphetamine OR “dextroamphetamine sulfate” OR “dexedrine spansule” OR procentra OR zenzedi) OR (adderall OR “adderall XR” OR mydayis) OR (“Sodium Oxybate”[Mesh] OR GHB OR “sodium oxybate”) AND (sleepiness OR “Narcolepsy”[Mesh] OR “excessive daytime sleepiness” OR narcolepsy)	1,343
Web of science	((solriamfetol OR JZP-110 OR ADX-N05 OR Sunosi) OR (modafinil OR provigil) OR (armodafinil OR nuvigil) OR (pitolisant OR wakix) OR (methylphenidate OR “methylphenidate hydrochloride” OR ritalin OR “ritalin LA” OR concerta) OR (amphetamine OR “amphetamine sulfate” OR adzenys OR dyanavel OR evekeo) OR (dextroamphetamine OR “dextroamphetamine sulfate” OR “dexedrine spansule” OR procentra OR zenzedi) OR (adderall OR “adderall XR” OR mydayis) OR (“sodium oxybate” OR “sodium oxybutyrate” OR GHB OR “sodium gamma-hydroxybutyrate” OR “sodium gamma hydroxybutyrate” OR “gamma-hydroxybutyrate” OR “gamma hydroxybutyrate”)) AND (sleepiness OR narcolepsy OR “excessive daytime sleepiness” OR “paroxysmal sleep” OR “narcoleptic syndrome” OR “narcoleptic syndromes” OR “gelineau syndrome” OR “gelineau's Syndrome” OR “gelineau's syndromes” OR “gelineaus syndrome” OR “narcolepsy-cataplexy syndrome” OR “narcolepsy cataplexy syndrome” OR “narcolepsy-cataplexy syndromes”)	1,775
CENTRAL	((solriamfetol OR JZP-110 OR ADX-N05 OR Sunosi) OR (modafinil OR provigil) OR (armodafinil OR nuvigil) OR (pitolisant OR wakix) OR (methylphenidate OR “methylphenidate hydrochloride” OR ritalin OR “ritalin LA” OR concerta) OR (amphetamine OR “amphetamine sulfate” OR adzenys OR dyanavel OR evekeo) OR (dextroamphetamine OR “dextroamphetamine sulfate” OR “dexedrine spansule” OR procentra OR zenzedi) OR (adderall OR “adderall XR” OR mydayis) OR (“sodium oxybate” OR “sodium oxybutyrate” OR GHB OR “sodium gamma-hydroxybutyrate” OR “sodium gamma hydroxybutyrate” OR “gamma-hydroxybutyrate” OR “gamma hydroxybutyrate”)) AND (sleepiness OR narcolepsy OR “excessive daytime sleepiness” OR “paroxysmal sleep” OR “narcoleptic syndrome” OR “narcoleptic syndromes” OR “gelineau syndrome” OR “gelineau's Syndrome” OR “gelineau's syndromes” OR “gelineaus syndrome” OR “narcolepsy-cataplexy syndrome” OR “narcolepsy cataplexy syndrome” OR “narcolepsy-cataplexy syndromes”)	637

Supplementary Table S1 (continued)

Database	Search terms	Result
Embase	((solriamfetol OR JZP-110 OR ADX-N05 OR Sunosi) OR (modafinil OR provigil) OR (armodafinil OR nuvigil) OR (pitolisant OR wakix) OR (methylphenidate OR “methylphenidate hydrochloride” OR ritalin OR “ritalin LA” OR concerta) OR (amphetamine OR “amphetamine sulfate” OR adzenys OR dyanavel OR evekeo) OR (dextroamphetamine OR “dextroamphetamine sulfate” OR “dexedrine spansule” OR procentra OR zenzedi) OR (adderall OR “adderall XR” OR mydayis) OR (“sodium oxybate” OR “sodium oxybutyrate” OR GHB OR “sodium gamma-hydroxybutyrate” OR “sodium gamma hydroxybutyrate” OR “gamma-hydroxybutyrate” OR “gamma hydroxybutyrate”)) AND (sleepiness OR narcolepsy OR “excessive daytime sleepiness” OR “paroxysmal sleep” OR “narcoleptic syndrome” OR “narcoleptic syndromes” OR “gelineau syndrome” OR “gelineau's Syndrome” OR “gelineau's syndromes” OR “gelineaus syndrome” OR “narcolepsy-cataplexy syndrome” OR “narcolepsy cataplexy syndrome” OR “narcolepsy-cataplexy syndromes”) AND ('clinical trial'/de OR 'human'/de) AND 'controlled study'/de	887
Clinical Trials.gov	Narcolepsy (intervention)	71
CNKI	嗜睡症 OR 發作性睡病 AND 臨床	254
EBSCO	((solriamfetol OR JZP-110 OR ADX-N05 OR Sunosi) OR (modafinil OR provigil) OR (armodafinil OR nuvigil) OR (pitolisant OR wakix) OR (methylphenidate OR “methylphenidate hydrochloride” OR ritalin OR “ritalin LA” OR concerta) OR (amphetamine OR “amphetamine sulfate” OR adzenys OR dyanavel OR evekeo) OR (dextroamphetamine OR “dextroamphetamine sulfate” OR “dexedrine spansule” OR procentra OR zenzedi) OR (adderall OR “adderall XR” OR mydayis) OR (“sodium oxybate” OR “sodium oxybutyrate” OR GHB OR “sodium gamma-hydroxybutyrate” OR “sodium gamma hydroxybutyrate” OR “gamma-hydroxybutyrate” OR “gamma hydroxybutyrate”)) AND (sleepiness OR narcolepsy OR “excessive daytime sleepiness” OR “paroxysmal sleep” OR “narcoleptic syndrome” OR “narcoleptic syndromes” OR “gelineau syndrome” OR “gelineau's Syndrome” OR “gelineau's syndromes” OR “gelineaus syndrome” OR “narcolepsy-cataplexy syndrome” OR “narcolepsy cataplexy syndrome” OR “narcolepsy-cataplexy syndromes”)	79

CENTRAL=Cochrane Central Register of Controlled trials; CNKI=China National Knowledge Infrastructure; EBSCO=Psychology and Behavioral Sciences Collection

Due to the lack of literature regarding amphetamine-type stimulants, they were not included in further analyses.

Supplementary Table S2 – **Categories of adverse events**

**Type of adverse events**	**Example**	**Study**
Gastrointestinal adverse event	Abdominal discomfort, dyspepsia, diarrhea, constipation, nausea, vomiting, dry mouth, anorexia, decreased appetite	15 studies[1-15]
Immunologic adverse event	Nasopharyngitis, rhinitis, upper respiratory tract infection, Influenza, urinary tract infection, infection	9 studies[1, 4, 6-9, 11, 13, 14]
Musculoskeletal adverse event	Myalgia, back pain	5 studies[1, 2, 8, 9, 11]
Neurological adverse event	Headache, dizziness, tremor	15 studies[1-15]
Psychological adverse event	Anxiety, nervousness, irritability, apathy, mood change, depression	10 studies[2-6, 8-10, 13, 14]
Sleep-related adverse event	Insomnia	6 studies[1-5, 8]
Other adverse event	Paresthesia, hypothermia	2 studies[9, 13]
Serious adverse event		8 studies[1, 3-7, 12, 14]
Adverse event resulting in participant withdrawal		11 studies[1, 3-10, 12, 13]
Any adverse event		7 studies[1-6, 13]

Supplementary Table S3 – **Reasons for exclusion**

**Study**	**Reason for exclusion**
Miller 1999 [16]	Commentary - Not RCT
Black 2003 [17]	Open-label - Not RCT
Rosenberg 2021 [18]	Post-hoc analysis - Not RCT
Doghramji 2019 [19]	Inappropriate primary outcome - Pharmacokinetics
Patricia 2019 [20]	Inappropriate population - Population includes idiopathic hypersomnia
Thorpy 2020 [21]	Corrigendum - results published in a full paper. See Thorpy 2019
Dauvilliers 2020 [22]	Post-hoc analysis. See Thorpy 2019;<br>Inappropriate primary outcome
Arnulf 2004 [23]	Editorial - Not RCT
Bauer 2019 [24]	Open-label - Not RCT
Bauer 2020 [25]	Open-label - Not RCT
Beusterien 1999 [26]	Inappropriate primary outcome - health-related quality-of-life (SF-36)
Billiard 1994 [27]	Inappropriate primary outcome
Black 2015 [28]	Secondary data analysis - Not RCT
Black 2004 [29]	Data had previously published. See Black et al, 2002
Black 2016 [30]	Secondary data analysis - Not RCT
Black 2014 [31]	Abstract - results published in a full paper. See Ruoff 2016
Black 2015 [32]	Abstract - results published in a full paper. See Ruoff 2016
Black 2014 [33]	Abstract - results published in a full paper. See Ruoff 2016
Black 2010 [34]	Open-label - Not RCT
Blackman 2001 [35]	Inappropriate intervention
Boero 2006 [36]	Abstract - results published in a full paper. See Mamelak 2004
Bogan 2019 [37]	Abstract - results published in a full paper. See Bogan 2020
Bogan 2005 [38]	Inappropriate primary outcome - sleep architecture
Bogan 2014 [39]	Abstract - results published in a full paper. See Bogan 2015
Bogan 2013 [40]	Abstract - results published in a full paper. See Bogan 2015
Bogan 2015 [41]	Post-hoc analysis - Not RCT
Broughton 1997 [42]	Inappropriate primary outcome
Causse 2017 [43]	Abstract - results published in a full paper. See Szakacs et 2017
Borgharkar 2010 [44]	Insufficient data
Dauvilliers 2014 [45]	Abstract - results published in a full paper. See Dauvilliers 2013
Dauvilliers 2019 [46]	Abstract - results published in a full paper. See Dauvilliers 2019
Dauvilliers 2016 [47]	Abstract - results published in a full paper. See Dauvilliers 2019
Dauvilliers 2019 [48]	Open-label - Not RCT
Dauvilliers 2018 [49]	Abstract - results published in a full paper. See Dauvilliers 2019
Dauvilliers 2017 [50]	Abstract - results published in a full paper. See Dauvilliers 2019
Dauvilliers 2019 [51]	Abstract - results published in a full paper. See Dauvilliers 2019
Dauvilliers 2020 [52]	Abstract - results published in a full paper. See Bogan 2021
Dauvilliers 2007 [53]	Inappropriate population
Dauvilliers 2013 [54]	Inappropriate primary outcome - nighttime sleep quality
Dauvilliers 2018 [55]	Abstract - results published in a full paper. See Dauvilliers 2020
Dauvilliers 2019 [56]	Abstract - results published in a full paper. See Bogan 2021
Dauvilliers 2016 [57]	Abstract - results published in a full paper. See Szakacs 2017
Davis 2020 [58]	Post-hoc analysis - Not RCT

Duntley 2005 [59]	Inappropriate primary outcome
Emsellem 2018 [60]	Abstract - results published in a full paper. See Emsellem 2020
Emsellem 2018 [61]	Abstract - results published in a full paper. See Emsellem 2020
Emsellem 2000 [62]	Open-label - Not RCT
Bassetti 2010 [63]	Insufficient data
Avadel 2016 [64]	Insufficient data
Plazzi 2018 [65]	Inappropriate population
Plazzi 2015 [66]	Inappropriate population
Feldman 2005 [67]	Insufficient data
Feldman 2001 [68]	Insufficient data
Foldvary-Schaefer 2020 [69]	Abstract - results published in a full paper. See Bogan 2021
Foldvary-Schaefer 2020 [70]	Abstract - results published in a full paper. See Bogan 2021
Foldvary-Schaefer 2020 [71]	Abstract - results published in a full paper. See Malhotra 2020
Fry 1996 [72]	Abstract - results published in a full paper. See Fry 1998
Harsh 2005 [73]	Abstract - results published in a full paper. See Harsh 2006
Hayduk 2006 [74]	Insufficient data
Hayduk 2001 [75]	Insufficient data
Hidalgo 2016 [76]	Not RCT
Hirshkowitz 2004 [77]	Insufficient data
Hirshkowitz 2001 [78]	Not RCT
Hong 2000 [79]	Non-pharmacological intervention
Hull 2006 [80]	Insufficient data
Ivanenko 2017 [81]	Not RCT and inappropriate population
Joo 2008 [82]	Not RCT and inappropriate primary outcome
Kovačević-Ristanović 2010 [83]	Commentary - not RCT
Laffont 1994 [84]	Not RCT
Lammers 2010 [85]	Commentary - not RCT
Lavault 2011 [86]	Not RCT
Malhotra 2020 [87]	Not RCT
Malhotra 2018 [88]	Inappropriate population - Population who had completed prior studies with solriamfetol
Malhotra 2019 [89]	Inappropriate primary outcome - weight change
Malhotra 2020 [90]	Inappropriate primary outcome
Mamelak 2015 [91]	Open-label - Not RCT
Mamelak 2014 [92]	Open-label - Not RCT
Mayer 2010 [93]	Not RCT and inappropriate primary outcome
Mayer 2011 [94]	Not RCT and inappropriate primary outcome
Meskill 2020 [95]	Commentary - not RCT
Mitler 1986 [96]	Not RCT
Montplaisir 2001 [97]	Insufficient data
Lee 2005 [98]	Insufficient data
Alza 2007 [99]	Insufficient data
Dauvilliers 2016 [100]	Insufficient data
Ramaekers 2016 [101]	Inappropriate primary outcome - Standard Deviation of Lateral Position (SDLP)

Trotti 2018 [102]	Inappropriate population
Nicollet 2000 [103]	Not RCT
Parkes 1973 [104]	Inappropriate primary outcome - Narcolepsy attacks
Pepin 2019 [105]	Abstract - results published in a full paper. See Malhotra 2020
Pepin 2018 [106]	Abstract - results published in a full paper. See Malhotra 2020
Philip 2014 [107]	Inappropriate population - IH
Richter 1997 [108]	Abstract - results published in a full paper. See Gross 2000
Rosenberg 2020 [109]	Post-hoc analysis - Not RCT
Rosenberg 2020 [110]	Abstract - results published in a full paper. See Rosenberg 2020
Rosenberg 2020 [111]	Abstract - results published in a full paper. See Rosenberg 2020
Rosenberg 2018 [112]	Abstract - results published in a full paper. See Rosenberg 2020
Rosenberg 2009 [113]	Abstract - results published in a full paper. See Harsh 2006
Rosenberg 2019 [114]	No usable data
Rosenberg 2006 [115]	Insufficient data
Roth 2013 [116]	Inappropriate primary outcome - Sleep quality
Roth 2009 [117]	Abstract - results published in a full paper. See Harsh 2006
Roth 2008 [118]	Abstract - results published in a full paper. See Harsh 2006
Roy 2020 [119]	Secondary data analysis - Not RCT
Ruoff 2016 [120]	Abstract - results published in a full paper. See Ruoff 2017
Ruoff 2016 [121]	Abstract - results published in a full paper. See Ruoff 2017
Ruoff 2017 [122]	Post-hoc analysis - Not RCT
Sagaspe 2017 [123]	Inappropriate population - IH
Sagaspe 2018 [124]	Inappropriate population - IH
Sahota 1997 [125]	Insufficient data
Saletu 2007 [126]	No usable data
Scharf 2001 [127]	Insufficient data
Schwartz 2004 [128]	Open-label - Not RCT
Schwartz 2003 [129]	Insufficient data
Schwartz 2003 [130]	Abstract - results published in a full paper. See Schwartz 2003
Schweitzer 2018 [131]	Secondary data analysis - Not RCT
Scrima 2015 [132]	Post-hoc analysis - Not RCT
Shapiro 2018 [133]	Inappropriate population; Abstract - results published in a full paper. See Malhotra 2018
Strollo 2020 [134]	Inappropriate primary outcome
Stultz 2020 [135]	Open-label - Not RCT
Szakacs 2017 [136]	Abstract - results published in a full paper. See Szakacs 2017
Takahashi 1979 [137]	Insufficient data
Thorpy 2004 [138]	Commentary - not RCT
Thorpy 2017 [139]	Abstract - results published in a full paper. See Thorpy 2019 and Emsellem 2020
Thorpy 2017 [140]	Abstract - results published in a full paper. See Thorpy 2019
Thorpy 2017 [141]	Abstract - results published in a full paper. See Thorpy 2019
Thorpy 2019 [142]	Secondary data analysis - Not RCT
Thorpy 2020 [143]	Abstract - results published in a full paper. See Bogan 2021
Thorpy 2018 [144]	Abstract - results published in a full paper. See Thorpy 2019
Heide 2015 [145]	Secondary data analysis - Not RCT
Villa 2015 [146]	Abstract - results published in a full paper. See Villa 2015
Vinckenbosch 2020 [147]	Inappropriate primary outcome
Weaver 2005 [148]	Abstract - results published in a full paper. See Weaver 2006

Weaver 2006 [149]	Inappropriate primary outcome - Functional Outcomes of Sleep Questionnaire (FOSQ)
Weaver 2018 [150]	Abstract - results published in a full paper. See Emsellem 2020
Wesnes 2005 [151]	Inappropriate primary outcome
Wesnes 2005 [152]	Abstract - results published in a full paper. See Wesnes 2005
Winter 2020 [153]	Inappropriate primary outcome
Yan 2013 [154]	Inappropriate primary outcome
Scrima 1990 [155]	Inappropriate primary outcome
Mitler 1993 [156]	Inappropriate primary outcome and intervention
Black 2009 [157]	Inappropriate primary outcome
Black 2010 [158]	Inappropriate primary outcome
Schwartz 2004 [159]	Inappropriate intervention
Schwartz 2003 [160]	Inappropriate intervention
Dauvilliers 2013 (Harmony 4) [161]	No usable data
Emsellem 2020 [162]	Inappropriate primary outcome
Saletu 2005 [163]	Inappropriate primary outcome

Supplementary Table S4-Summary of trials and pharmacological interventions that included in the network meta-analysis.

Study	Area; study design	Intervention	Control	Number of participants (intervention; control)	Age, years (intervention; control)	Gender of participants (intervention; control)	Diagnoses	ESS change	MWT change	Cataplexy rate change	CGI-C	AE
Bogan et al, 2021	USA, Europe; parallel; RWD	Lower-sodium oxybate (3-9 g), equally divided at bedtime and 2.5-4 hours later	Placebo	69; 65	37.2 (11.79); 37.8 (12.69)	Male, 26/69 (37.7%), female, 43/69 (62.3%); male, 26/65 (40.0%), female, 39/65 (60.0%)	Narcolepsy with cataplexy	+		+ (weekly)	+	+
Bogan et al, 2015	USA; crossover	Solriamfetol (150 mg per day for 7 days followed by 300 mg per day for 7 days before breakfast)	Placebo	33	37.1 (12.4)	Male, 19/33 (57.6%), female, 14/33 (42.4%)	Narcolepsy with or without cataplexy	+	+ (40-min)		+	+
Thorpy et al, 2019	USA, Canada, Europe; parallel	Solriamfetol (75 mg per day) vs solriamfetol (150 mg per day) vs solriamfetol (300 mg per day)	Placebo	59; 55; 59; 58	36.5 (12.78); 38.1 (13.00); 34.3 (11.51); 36.0 (15.17)	Male, 22/59 (37.3%), female, 37/59 (62.7%); male, 17/59 (28.8%), female, 42/59 (71.2%); male, 19/59 (32.2%), female, 40/59 (67.8%); male, 24/59 (40.7%), female, 35/59 (59.3%)	Narcolepsy with or without cataplexy	+	+ (40-min)		+	+
Ruoff et al, 2016	USA; parallel	Solriamfetol (150 mg per day for 4 weeks followed by 300 mg per day for 8 weeks before breakfast)	Placebo	43; 47	41.0 (12.3); 36.7 (11.7)	Male, 14/44 (31.8%), female, 30/44 (68.2%); male, 19/49 (38.8%), female, 30/49 (61.2%)	Narcolepsy with or without cataplexy	+	+ (40-min)		+	+

Supplementary Table S4 (continued)

Study	Area; study design	Intervention	Control	Number of participants (intervention; control)	Age, years (intervention; control)	Gender of participants (intervention; control)	Diagnoses	ESS change	MWT change	Cataplexy rate change	CGI-C	AE
Dauvilliers et al, 2013 (Harmony I)	Europe; parallel	Pitolisant (10 or 20 or 40 mg per day before lunch)	Modafinil (100 or 200 or 400 mg per day before breakfast) vs placebo	31; 33; 30	Median (IQR): 33.0 (21-49); 40.0 (25-48); 39.5 (30-52)	Male, 20/31 (65%), female, 11/31 (35%); male, 18/33 (55%), female, 15/33 (45%); male, 13/30 (43%), female, 17/30 (57%)	Narcolepsy with or without cataplexy	+	+ (20-min)	+ (daily)	+	+
Harmony Ibis, 2010 (reported by Kollb-Sielecka 2017)	Europe; parallel	Pitolisant (10 or 20 mg per day)	Modafinil (100 or 200 or 400 mg per day) vs placebo	67; 65; 33	N/A	N/A	Narcolepsy with or without cataplexy	+	+ (40-min)	+ (daily)		
Szakacs et al, 2017 (Harmony CTP)	Europe, Russia, Turkey; parallel	Pitolisant (5 or 10 or 20 or 40 mg before breakfast)	Placebo	54; 51	Median (range): 34 (18–64); 39 (18–66)	Male, 26/54 (48%), female, 28/51 (52%); male, 27/51 (53%), female, 24/51 (47%)	Narcolepsy with cataplexy	+	+ (40-min)	+ (weekly)	+	+
Harsh et al, 2006	USA, Canada, Europe, Australia, Russia; parallel	Armodafinil (150 mg per day) vs armodafinil (250 mg per day) 0800 before breakfast	Placebo	58; 60; 58	40.4 (12.5); 35.0 (12.5); 39.2 (12.0)	Male, 28/64 (44%), female, 36/64 (56%); male, 25/67 (37%), female, 42/67 (63%); male, 32/63 (51%), female, 31/63 (49%)	Narcolepsy with or without cataplexy		+ (20-min)	+ (daily)	+	+

Supplementary Table S4 (continued)

Study	Area; study design	Intervention	Control	Number of participants (intervention; control)	Age, years (intervention; control)	Gender of participants (intervention; control)	Diagnoses	ESS change	MWT change	Cataplexy rate change	CGI-C	AE
Moldofsky et al, 2000	Canada; parallel; RWD	Modafinil (300 ± 100 mg per day)	Placebo	30; 33	45 (16)	Male, 21/63 (33.3%); female, 42/63 (66.7%)	Narcolepsy with or without cataplexy	+	+ (40-min)			+
Fry et al, 1998 (US Modafinil in Narcolepsy Multicenter Study Group)	USA; parallel	Modafinil (200 mg per day at morning) vs modafinil (400 mg per day at morning) 30 to 45 minutes after breakfast	Placebo	95; 86; 86	Mean (range): 40 (18~67); 44 (19~67); 42 (18~68)	Male, 44/96 (46%), female, 52/96 (54%); male, 43/95 (45%), female, 52/95 (55%); male, 42/92 (46%), female, 50/92 (54%)	Narcolepsy with or without cataplexy	+	+ (20-min)		+	+
Gross et al, 2000 (US Modafinil in Narcolepsy Multicenter Study Group)	USA; parallel	Modafinil (100 mg per day for 7 days, then 200 mg per day at morning) vs modafinil (100 mg per day for 7 days, 200 mg for 1 day, then 400 mg per day at morning)	Placebo	83; 86; 88	Mean (range): 42 (18~67); 42 (18~66); 41 (17~66)	Male, 37/89 (42%), female, 52/89 (58%); male, 44/89 (49%), female, 45/89 (51%); male, 43/93 (46%), female, 50/93 (54%)	Narcolepsy with or without cataplexy	+	+ (20-min)		+	+
Saletu et, al 2004	Austria; crossover	Modafinil (200 mg at morning; 200 mg at noon)	Placebo	16	39.1 (13.3)	Male, 10/16 (62.5%), female, 6/16 (37.5%)	Narcolepsy with or without cataplexy					+

Supplementary Table S4 (continued)

Study	Area; study design	Intervention	Control	Number of participants (intervention; control)	Age, years (intervention; control)	Gender of participants (intervention; control)	Diagnoses	ESS change	MWT change	Cataplexy rate change	CGI-C	AE
Saletu et, al 2009	Austria; crossover	Modafinil (200 mg at morning; 200 mg at noon)	Placebo	15	38 (18)	Male, 7/15 (46.7%), female, 8/15 (53.3%)	Narcolepsy with or without cataplexy	+				
Ahmed et al, 2005a (Xyrem International Study Group)	USA, Canada, Europe; parallel	Sodium oxybate (4.5 g) vs sodium oxybate (6 g) vs sodium oxybate (9 g), equally divided at bedtime and 2.5-4 hours later	Placebo	64; 58; 47; 59	Mean (range): 40.5 (16~75)	Male, 79/228 (34.6%), female, 149/228 (65.4%)	Narcolepsy with cataplexy	+	+ (40-min)		+	
Ahmed et al, 2005b (Xyrem International Study Group)	USA, Canada, Europe; parallel	Sodium oxybate (4.5 g) vs sodium oxybate (6 g) vs sodium oxybate (9 g), equally divided at bedtime and 2.5-4 hours later	Placebo	64; 58; 47; 59	Mean (range): 40.5 (16~75)	Male, 79/228 (34.6%), female, 149/228 (65.4%)	Narcolepsy with cataplexy			+ (weekly)		+
Black et al, 2006	USA, Canada, Europe; parallel	Sodium oxybate (9g nightly) plus placebo modafinil vs sodium oxybate (9 g nightly) plus modafinil (200-600 mg per day)	Placebo sodium oxybate plus modafinil (200-600 mg per day) vs placebo sodium oxybate plus placebo modafinil	50; 54; 63; 55	35.1 ± 12.9; 38.9 ± 15.9; 38.9 ± 15.6; 41.0 ± 13.4	Male, 26/50 (52%), female, 24/50 (48%); male, 25/54 (46.3%), female, 29/54 (53.7%); male, 32/63 (50.8%), female, 31/63 (49.2%); male, 24/55 (43.6%), female, 31/55 (56.4%)	Narcolepsy with or without cataplexy		+ (20-min)		+	+

Supplementary Table S4 (continued)

Study	Area; study design	Intervention	Control	Number of participants (intervention; control)	Age, years (intervention; control)	Gender of participants (intervention; control)	Diagnoses	ESS change	MWT change	Cataplexy rate change	CGI-C	AE
Cook et al, 2002 (US Xyrem Multicenter Study Group)	USA; parallel	Sodium oxybate (3 g) vs sodium oxybate (6 g) vs sodium oxybate (9 g), equally divided at bedtime and 2.5-4 hours later	Placebo	34; 33; 35; 34	Mean: 43.1	Male, 57/136 (41.9%), female, 79/136 (58.1)	Narcolepsy with cataplexy	+		+ (weekly)	+	+
Lammers et al, 1993	Europe; crossover	Gamma-hydroxybutyrate (60 mg/kg/night), equally divided at bedtime and 4 hours later	Placebo	24	Mean (range): 36.0 (16-65)	Male, 13/24 (54.2%), female, 11/24 (45.8%)	Narcolepsy with cataplexy			+ (daily)		
Scrima et al, 1989	USA; crossover	Gamma-hydroxybutyrate (50 mg/kg), equally divided at bedtime and 3 hours later	Placebo	20	Male, 49.1 (12.7), female, 45.9 (14.5)	Male, 10/20 (50%), female, 10/20 (50%)	Narcolepsy with cataplexy			+ (daily)		+

ESS, Epworth sleepiness scale; MWT, Maintenance of wakefulness test; CGI-C, Clinical Global Impression of Change; AE, adverse event; RWD, randomized withdrawal design

+ indicates outcome of interest was reported by the corresponding trial.

Supplementary Figure S1 – Cochrane risk of bias appraisal

Study	D1	D2	D3	D4	D5	Overall
Bogan et al, 2021	+	+	+	+	+	+
Thorpy et al, 2019	+	+	+	+	+	+
Bogan et al, 2015	-	X	-	+	+	X
Ruoff et al, 2016	-	+	+	+	+	-
Dauvilliers et al, 2013	+	+	+	+	+	+
Dauvilliers 2010	-	+	-	+	+	-
Szakacs et al, 2017	+	+	+	+	+	+
Harsh et al, 2006	+	+	+	+	+	+
Moldofsky et al, 2000	+	-	-	+	-	-
Fry et al, 1998	+	+	-	+	-	-
Gross et al, 2000	+	+	-	+	-	-
Saletu et al, 2004	+	X	-	+	-	X
Saletu et al, 2009	+	X	-	+	-	X
Ahmed et al, 2005	-	+	-	+	+	-
Ahmed et al, 2005b	-	+	-	+	+	-
Black et al, 2006	-	+	-	+	+	-
Cook et al, 2002	-	+	-	+	-	-
Lammers et al, 1993	-	X	+	+	-	X
Scrima et al, 1989	+	X	-	+	-	X

Domains:
D1: Bias arising from the randomization process.
D2: Bias due to deviations from intended intervention.
D3: Bias due to missing outcome data.
D4: Bias in measurement of the outcome.
D5: Bias in selection of the reported result.

Judgement
X High
- Some concerns
+ Low

Change in Epworth Sleepiness Scale

Supplementary Figure S2 – Net heat plot: ESS change

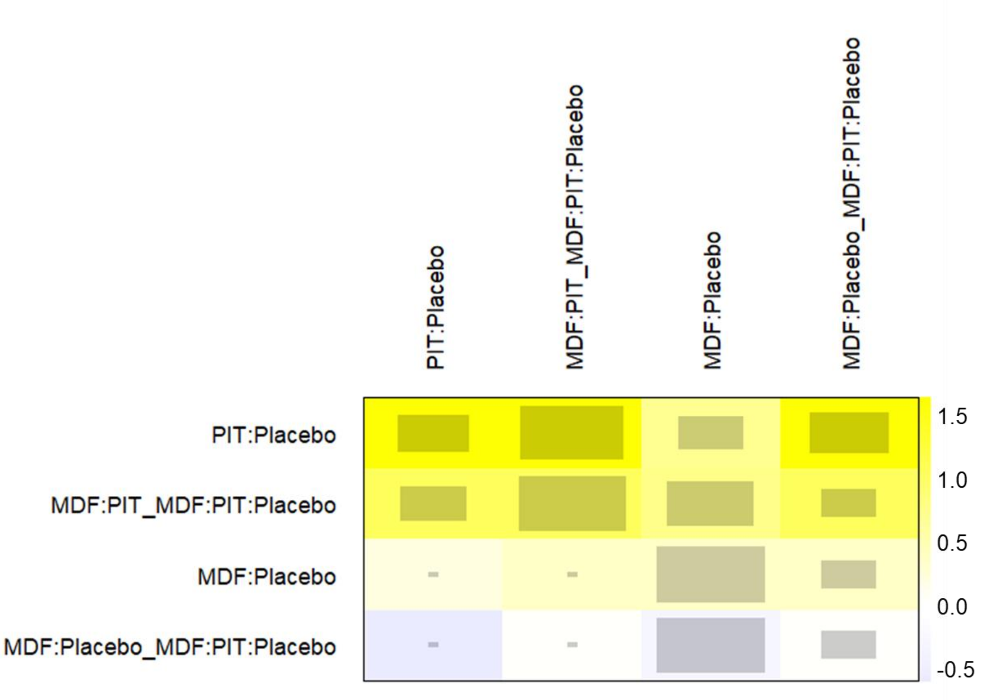

MDF=modafinil; PIT=pitolisant

Graphical display locates potential sources of inconsistency between direct and indirect evidence.

Inconsistency is represented as color, which red being the highest and blue the lowest.

The gray squares' area represents the contribution of the direct estimate of one design in the column to the network estimate in a row.

Pairwise comparisons within the three-arm designs are pointed out by "_".

Supplementary Table S5 – Assessment of inconsistency: ESS change

Comparison	k	Prop	NMA	Direct	Indir.	Diff	z	p-value
MDF:LXB	0	0	-0.3715	.	-0.3715	.	.	.
PIT:LXB	0	0	1.1186	.	1.1186	.	.	.
Placebo:LXB	1	1	3	3	.	.	.	.
SOF:LXB	0	0	-1.7647	.	-1.7647	.	.	.
SXB:LXB	0	0	0.7996	.	0.7996	.	.	.
MDF:PIT	2	0.63	-1.4902	-2.4995	0.2075	-2.707	-1.62	0.1046
MDF:Placebo	6	0.97	-3.3715	-3.1953	-8.3359	5.1406	1.76	0.079
MDF:SOF	0	0	1.3932	.	1.3932	.	.	.
MDF:SXB	0	0	-1.1711	.	-1.1711	.	.	.
PIT:Placebo	3	0.84	-1.8814	-2.3482	0.6401	-2.9883	-1.44	0.151
PIT:SOF	0	0	2.8833	.	2.8833	.	.	.
PIT:SXB	0	0	0.319	.	0.319	.	.	.
Placebo:SOF	3	1	4.7647	4.7647	.	.	.	.
Placebo:SXB	2	1	2.2004	2.2004	.	.	.	.
SOF:SXB	0	0	-2.5643	.	-2.5643	.	.	.

MDF=modafinil; LXB=lower-sodium oxybate; SXB=sodium oxybate; SOF=solriamfetol; PIT=pitolisant

Comparison: Treatment comparison
k: Number of studies providing direct evidence
Prop: Direct evidence proportion
NMA: Estimated treatment effect (MD) in network meta-analysis
Direct: Estimated treatment effect (MD) derived from direct evidence
Indir.: Estimated treatment effect (MD) derived from indirect evidence
Diff: Difference between direct and indirect treatment estimates
z: z-value of test for disagreement (direct versus indirect)
p-value: p-value of test for disagreement (direct versus indirect)

Supplementary Figure S3 – Funnel plot: ESS change

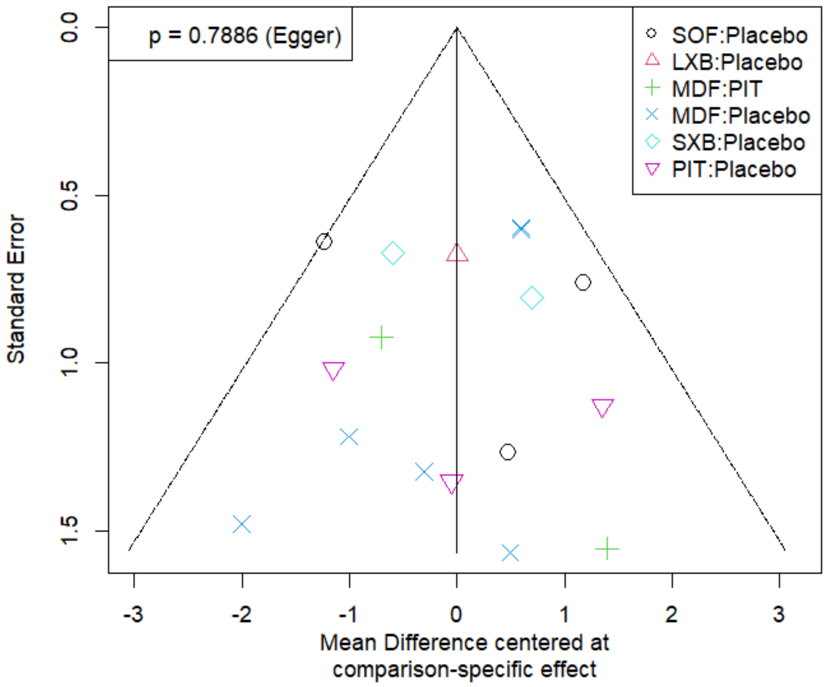

SOF=solriamfetol; LXB=lower-sodium oxybate; MDF=modafinil; PIT=pitolisant; SXB=sodium oxybate

Change in Epworth Sleepiness Scale – subgroup analyses

Supplementary Figure S4 – Network graph: Trials included participants with cataplexy (ESS change)

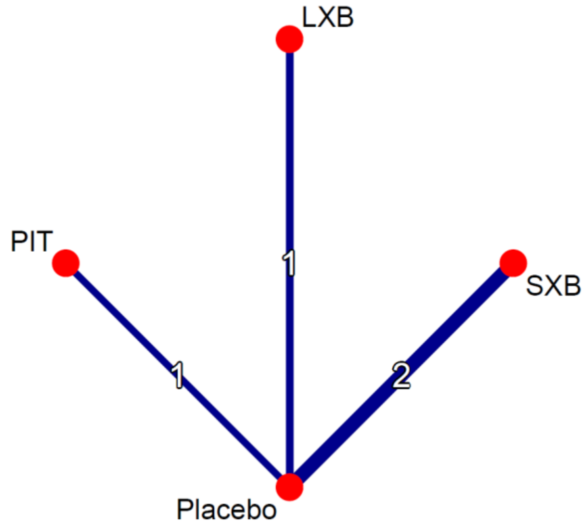

PIT=pitolisant; LXB=lower-sodium oxybate; SXB=sodium oxybate

Nodes: Interventions
Lines Head-to-head comparisons
The width of lines: Number of trials studying the comparison

Supplementary Table S6 – Assessment of inconsistency: Trials included participants with cataplexy (ESS change)

Comparison	k	Prop	NMA	Direct	Indir.	Diff	z	p-value
PIT:LXB	0	0	-0.5	.	-0.5	.	.	.
Placebo:LXB	1	1	3	3	.	.	.	.
SXB:LXB	0	0	0.7752	.	0.7752	.	.	.
PIT:Placebo	1	1	-3.5	-3.5	.	.	.	.
PIT:SXB	0	0	-1.2752	.	-1.2752	.	.	.
Placebo:SXB	2	1	2.2248	2.2248	.	.	.	.

PIT=pitolisant; LXB=lower-sodium oxybate; SXB=sodium oxybate

Comparison: Treatment comparison
k: Number of studies providing direct evidence
Prop: Direct evidence proportion
NMA: Estimated treatment effect (MD) in network meta-analysis
Direct: Estimated treatment effect (MD) derived from direct evidence
Indir.: Estimated treatment effect (MD) derived from indirect evidence
Diff: Difference between direct and indirect treatment estimates
z: z-value of test for disagreement (direct versus indirect)
p-value: p-value of test for disagreement (direct versus indirect)

Supplementary Figure S5 – Forest plot: Trials included participants with cataplexy (ESS change)

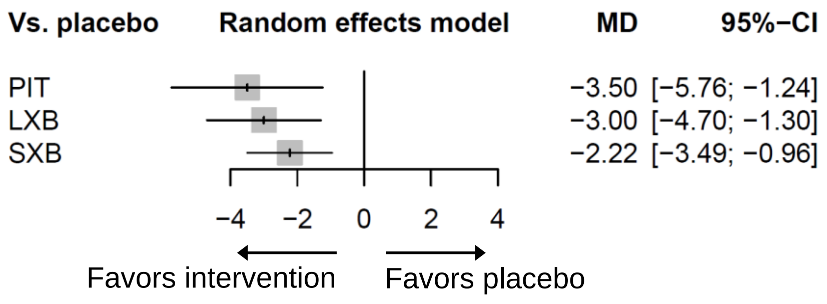

PIT=pitolisant; LXB=lower-sodium oxybate; SXB=sodium oxybate

Supplementary Table S7 – League table: Trials included participants with cataplexy (ESS change)

**Pitolisant**			
-0.50 (-3.33 - 2.33)	**Lower-sodium oxybate**		
-1.28 (-3.86 - 1.31)	-0.78 (-2.90 - 1.35)	**Sodium oxybate**	
**-3.50 (-5.76 - -1.24)**	**-3.00 (-4.70 - -1.30)**	**-2.22 (-3.49 - -0.96)**	**Placebo**

Interventions were ranked in a descending order of P-scores. Estimates for change in ESS were shown in mean difference with 95% confidence interval.

Change in Epworth Sleepiness Scale – sensitivity analysis

Supplementary Figure S6 – Forest plot: crossover design excluded (ESS change)

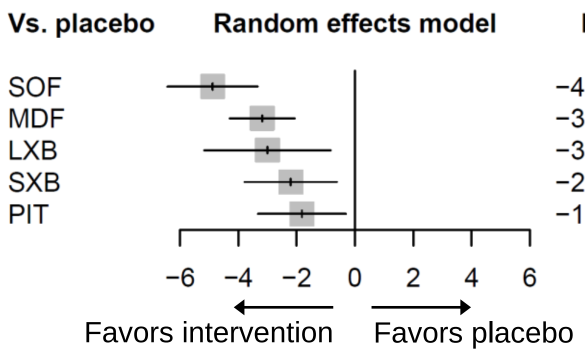

SOF=solriamfetol; MDF=modafinil; LXB=lower-sodium oxybate; SXB=sodium oxybate; PIT=pitolisant

Supplementary Table S8 – League table: crossover design excluded (ESS change)

**Solriamfetol**					
-1.70 (-3.62 - 0.21)	**Modafinil**				
-1.88 (-4.55 - 0.78)	-0.18 (-2.62 - 2.26)	**Lower-sodium oxybate**			
**-2.68 (-4.90 - -0.47)**	-0.98 (-2.92 - 0.96)	-0.80 (-3.49 - 1.88)	**Sodium oxybate**		
**-3.06 (-5.23 - -0.90)**	-1.36 (-2.99 - 0.26)	-1.18 (-3.83 - 1.46)	-0.38 (-2.57 - 1.81)	**Pitolisant**	
**-4.88 (-6.43 - -3.33)**	**-3.18 (-4.30 - -2.06)**	**-3.00 (-5.17 - -0.83)**	**-2.20 (-3.78 - -0.61)**	**-1.82 (-3.33 - -0.31)**	**Placebo**

Interventions were ranked in a descending order of P-scores. Estimates for change in ESS were shown in mean difference with 95% confidence interval.

Supplementary Figure S7 – Forest plot: withdrawal design excluded (ESS change)

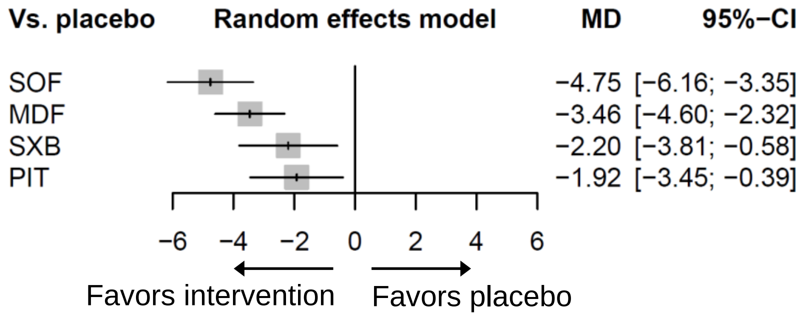

SOF=solriamfetol; MDF=modafinil; SXB=sodium oxybate; PIT=pitolisant

Supplementary Table S9 – League table: withdrawal design excluded (ESS change)

**Solriamfetol**				
-1.29<br>(-3.10 - 0.51)	**Modafinil**			
**-2.56<br>(-4.70 - -0.42)**	-1.26<br>(-3.24 - 0.71)	**Sodium oxybate**		
**-2.83<br>(-4.91 - -0.76)**	-1.54<br>(-3.19 - 0.11)	-0.28<br>(-2.50 - 1.95)	**Pitolisant**	
**-4.75<br>(-6.16 - -3.35)**	**-3.46<br>(-4.60 - -2.32)**	**-2.20<br>(-3.81 - -0.58)**	**-1.92<br>(-3.45 - -0.39)**	**Placebo**

Interventions were ranked in a descending order of P-scores. Estimates for change in ESS were shown in mean difference with 95% confidence interval.

Change in Maintenance of Wakefulness Test

Supplementary Figure S8 – Net heat plot: MWT change

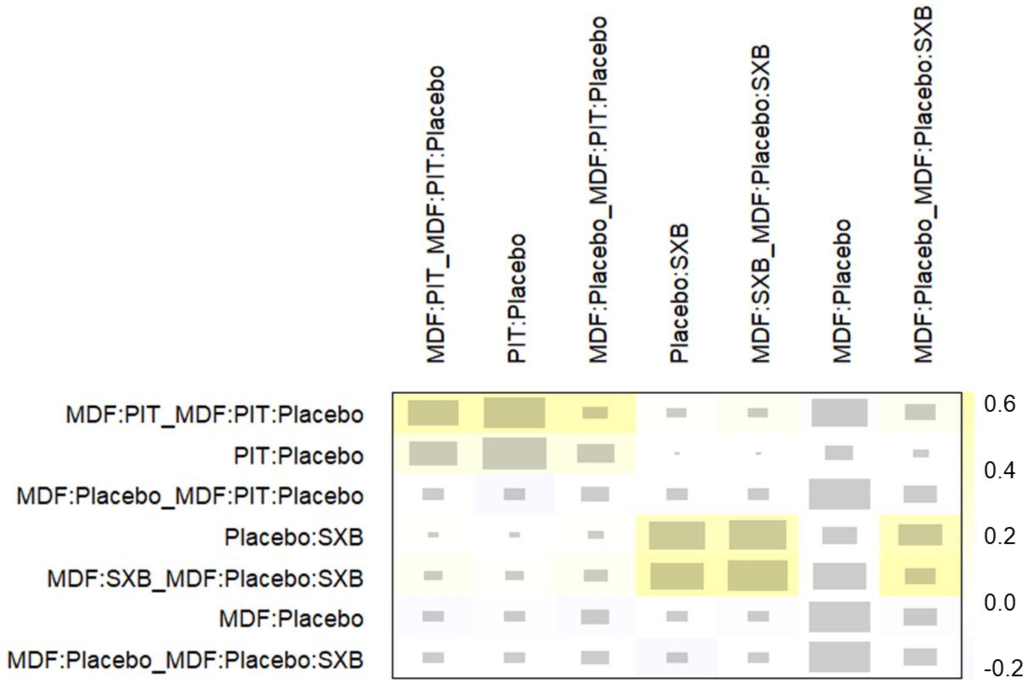

MDF=modafinil; PIT=pitolisant; SXB=sodium oxybate

Graphical display locates potential sources of inconsistency between direct and indirect evidence.

Inconsistency is represented as color, which red being the highest and blue the lowest.

The gray squares' area represents the contribution of the direct estimate of one design in the column to the network estimate in a row.

Pairwise comparisons within the three-arm designs are pointed out by "_".

Supplementary Table S10 – Assessment of inconsistency: MWT change

Comparison	k	Prop	NMA	Direct	Indir.	Diff	z	p-value
AMD:MDF	0	0	0.0294	.	0.0294	.	.	.
AMD:PIT	0	0	0.063	.	0.063	.	.	.
AMD:Placebo	1	1	0.584	0.584	.	.	.	.
AMD:SOF	0	0	-0.3892	.	-0.3892	.	.	.
AMD:SXB	0	0	0.0318	.	0.0318	.	.	.
MDF:PIT	1	0.35	0.0336	0.2797	-0.0971	0.3768	1.0039	0.3154
MDF:Placebo	5	0.94	0.5545	0.5602	0.4697	0.0905	0.2012	0.8405
MDF:SOF	0	0	-0.4186	.	-0.4186	.	.	.
MDF:SXB	1	0.5	0.0024	-0.2276	0.2301	-0.4576	-1.2732	0.203
PIT:Placebo	3	0.92	0.5209	0.5563	0.1068	0.4495	0.7762	0.4376
PIT:SOF	0	0	-0.4522	.	-0.4522	.	.	.
PIT:SXB	0	0	-0.0312	.	-0.0312	.	.	.
Placebo:SOF	3	1	-0.9731	-0.9731	.	.	.	.
Placebo:SXB	2	0.9	-0.5522	-0.4997	-1.0009	0.5012	0.9351	0.3497
SOF:SXB	0	0	0.421	.	0.421	.	.	.

AMD=armodafinil; MDF=modafinil; PIT=pitolisant; SOF=solriamfetol; SXB=sodium oxybate

Comparison: Treatment comparison
k: Number of studies providing direct evidence
Prop: Direct evidence proportion
NMA: Estimated treatment effect (SMD) in network meta-analysis
Direct: Estimated treatment effect (SMD) derived from direct evidence
Indir.: Estimated treatment effect (SMD) derived from indirect evidence
Diff: Difference between direct and indirect treatment estimates
z: z-value of test for disagreement (direct versus indirect)
p-value: p-value of test for disagreement (direct versus indirect)

Supplementary Figure S9 – Funnel plot: MWT change

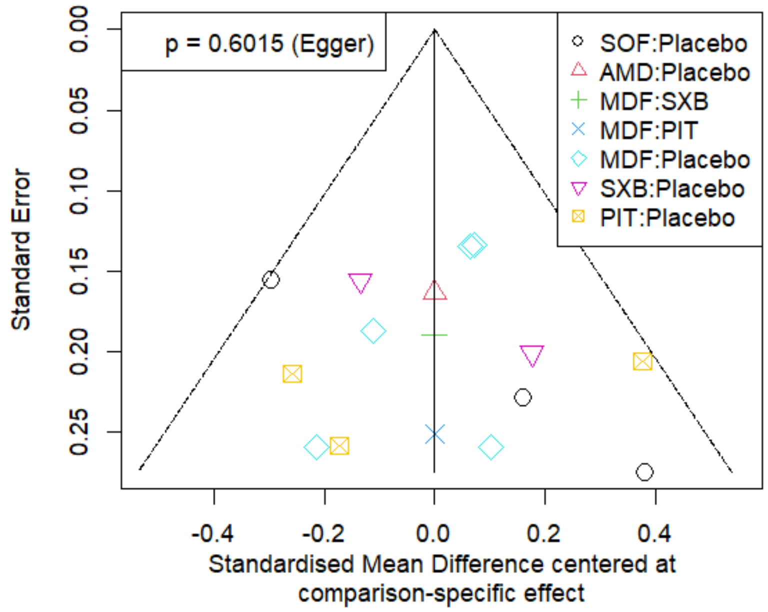

SOF=solriamfetol; AMD=armodafinil; MDF=modafinil; PIT=pitolisant; SXB=sodium oxybate

Change in Maintenance of Wakefulness Test – subgroup analyses

Supplementary Figure S10 – Network graph: Trials included participants with cataplexy (MWT change)

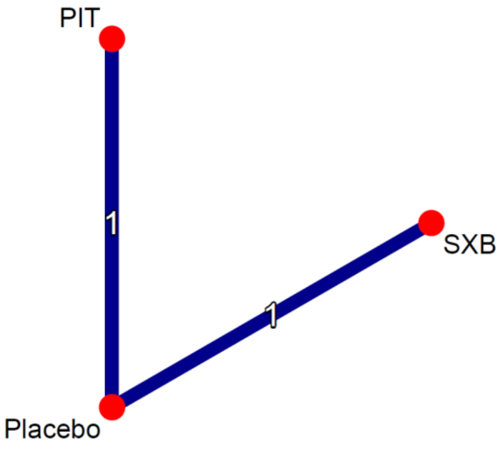

PIT=pitolisant; SXB=sodium oxybate

Nodes: Interventions

Lines Head-to-head comparisons

The width of lines: Number of trials studying the comparison

Supplementary Table S11 – Assessment of inconsistency: Trials included participants with cataplexy (MWT change)

Comparison	k	Prop	NMA	Direct	Indir.	Diff	z	p-value
PIT:Placebo	1	1	0.9325	0.9325	.	.	.	.
PIT:SXB	0	0	0.5681	.	0.5681	.	.	.
Placebo:SXB	1	1	-0.3644	-0.3644	.	.	.	.

PIT=pitolisant; SXB=sodium oxybate

Comparison: Treatment comparison
k: Number of studies providing direct evidence
Prop: Direct evidence proportion
NMA: Estimated treatment effect (SMD) in network meta-analysis
Direct: Estimated treatment effect (SMD) derived from direct evidence
Indir.: Estimated treatment effect (SMD) derived from indirect evidence
Diff: Difference between direct and indirect treatment estimates
z: z-value of test for disagreement (direct versus indirect)
p-value: p-value of test for disagreement (direct versus indirect)

Supplementary Figure S11 – Forest plot: Trials included participants with cataplexy (MWT change)

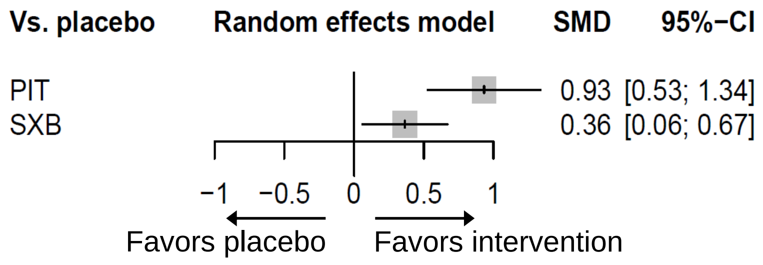

PIT=pitolisant; SXB=sodium oxybate

Supplementary Table S12 – League table: Trials included participants with cataplexy (MWT change)

**Pitolisant**		
**0.57 (0.06 - 1.07)**	**Sodium oxybate**	
**0.93 (0.53 - 1.34)**	**0.36 (0.06 - 0.67)**	**Placebo**

Interventions were ranked in a descending order of P-scores. Estimates for change in MWT were shown in standardized mean difference with 95% confidence interval.

Change in Maintenance of Wakefulness Test – sensitivity analysis

Supplementary Figure S12 – Forest plot: crossover design excluded (MWT change)

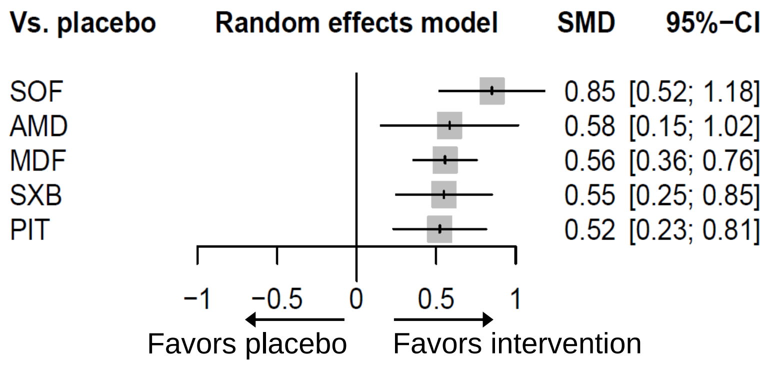

SOF=solriamfetol; AMD=armodafinil; MDF=modafinil; SXB=sodium oxybate; PIT=pitolisant

Supplementary Table S13 – League table: crossover design excluded (MWT change)

**Solriamfetol**					
0.27 (-0.28 - 0.81)	**Armodafinil**				
0.29 (-0.09 - 0.68)	0.03 (-0.45 - 0.50)	**Modafinil**			
0.30 (-0.15 - 0.75)	0.03 (-0.49 - 0.56)	0.01 (-0.32 - 0.34)	**Sodium oxybate**		
0.33 (-0.11 - 0.77)	0.06 (-0.46 - 0.58)	0.03 (-0.30 - 0.37)	0.03 (-0.39 - 0.44)	**Pitolisant**	
**0.85 (0.52 - 1.18)**	**0.58 (0.15 - 1.02)**	**0.56 (0.36 - 0.76)**	**0.55 (0.25 - 0.85)**	**0.52 (0.23 - 0.81)**	**Placebo**

Interventions were ranked in a descending order of P-scores. Estimates for change in MWT were shown in standardized mean difference with 95% confidence interval.

Supplementary Figure S13 – Forest plot: withdrawal design excluded (MWT change)

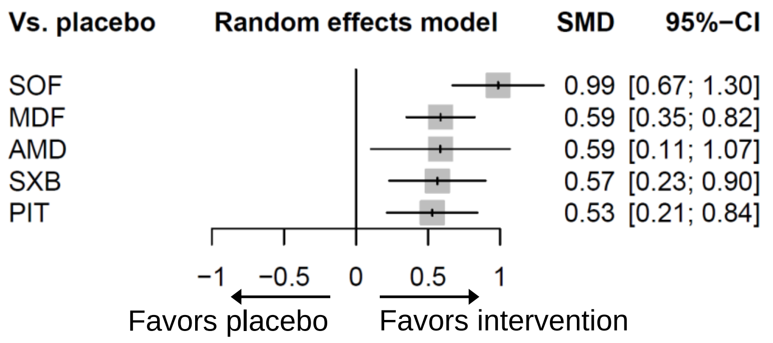

SOF=solriamfetol; MDF=modafinil; AMD=armodafinil; SXB=sodium oxybate; PIT=pitolisant

Supplementary Table S14 – League table: withdrawal design excluded (MWT change)

**Solriamfetol**					
0.40 (0.00 - 0.80)	**Modafinil**				
0.40 (-0.18 - 0.98)	0.00 (-0.54 - 0.54)	**Armodafinil**			
0.42 (-0.04 - 0.88)	0.02 (-0.35 - 0.39)	0.02 (-0.56 - 0.61)	**Sodium oxybate**		
**0.46 (0.01 - 0.90)**	0.06 (-0.31 - 0.43)	0.06 (-0.52 - 0.63)	0.04 (-0.42 - 0.49)	**Pitolisant**	
**0.99 (0.67 - 1.30)**	**0.59 (0.35 - 0.82)**	**0.59 (0.11 - 1.07)**	**0.57 (0.23 - 0.90)**	**0.53 (0.21 - 0.84)**	**Placebo**

Interventions were ranked in a descending order of P-scores. Estimates for change in MWT were shown in standardized mean difference with 95% confidence interval.

Change in cataplexy rate

Supplementary Table S15 – Assessment of inconsistency: cataplexy rate change

Comparison	k	Prop	NMA	Direct	Indir.	Diff	z	p-value
AMD:LXB	0	0	0.6361	.	0.6361	.	.	.
AMD:MDF	0	0	0.1982	.	0.1982	.	.	.
AMD:PIT	0	0	0.7244	.	0.7244	.	.	.
AMD:Placebo	1	1	0	0	.	.	.	.
AMD:SXB	0	0	0.4001	.	0.4001	.	.	.
LXB:MDF	0	0	-0.438	.	-0.438	.	.	.
LXB:PIT	0	0	0.0882	.	0.0882	.	.	.
LXB:Placebo	1	1	-0.6361	-0.6361	.	.	.	.
LXB:SXB	0	0	-0.236	.	-0.236	.	.	.
MDF:PIT	1	0.8	0.5262	0.7015	-0.1939	0.8954	1.0556	0.2912
MDF:Placebo	1	0.81	-0.1982	-0.3691	0.5367	-0.9059	-1.0556	0.2912
MDF:SXB	0	0	0.202	.	0.202	.	.	.
PIT:Placebo	3	1	-0.7244	-0.7243	.	.	.	.
PIT:SXB	0	0	-0.3242	.	-0.3242	.	.	.
Placebo:SXB	4	1	0.4001	0.4001	.	.	.	.

AMD=armodafinil; LXB=lower-sodium oxybate; MDF=modafinil; PIT=pitolisant; SXB=sodium oxybate

Comparison: Treatment comparison
k: Number of studies providing direct evidence
Prop: Direct evidence proportion
NMA: Estimated treatment effect (SMD) in network meta-analysis
Direct: Estimated treatment effect (SMD) derived from direct evidence
Indir.: Estimated treatment effect (SMD) derived from indirect evidence
Diff: Difference between direct and indirect treatment estimates
z: z-value of test for disagreement (direct versus indirect)
p-value: p-value of test for disagreement (direct versus indirect)

Change in cataplexy rate – subgroup analyses

Supplementary Figure S14 –Network graph: FDA approved Interventions for cataplexy (cataplexy rate change)

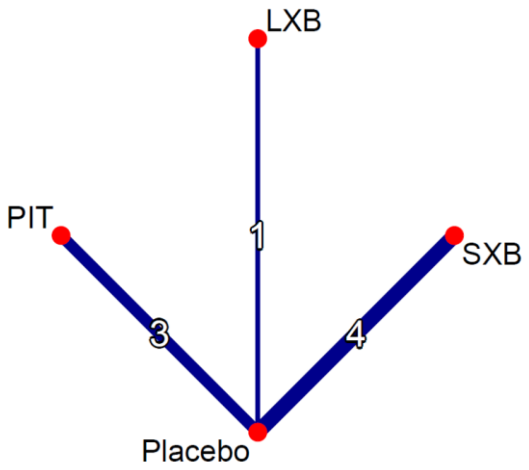

LXB=lower-sodium oxybate; PIT=pitolisant; SXB=sodium oxybate

Nodes: Interventions

Lines Head-to-head comparisons

The width of lines: Number of trials studying the comparison

Supplementary Table S16 – Assessment of inconsistency: FDA approved Interventions for cataplexy (cataplexy rate change)

Comparison	k	Prop	NMA	Direct	Indir.	Diff	z	p-value
LXB:PIT	0	0	0.0409	.	0.0409	.	.	.
LXB:Placebo	1	1	-0.6361	-0.6361	.	.	.	.
LXB:SXB	0	0	-0.2379	.	-0.2379	.	.	.
PIT:Placebo	3	1	-0.677	-0.677	.	.	.	.
PIT:SXB	0	0	-0.2787	.	-0.2787	.	.	.
Placebo:SXB	4	1	0.3982	0.3982	.	.	.	.

LXB=lower-sodium oxybate; PIT=pitolisant; SXB=sodium oxybate

Comparison: Treatment comparison
k: Number of studies providing direct evidence
Prop: Direct evidence proportion
NMA: Estimated treatment effect (SMD) in network meta-analysis
Direct: Estimated treatment effect (SMD) derived from direct evidence
Indir.: Estimated treatment effect (SMD) derived from indirect evidence
Diff: Difference between direct and indirect treatment estimates
z: z-value of test for disagreement (direct versus indirect)
p-value: p-value of test for disagreement (direct versus indirect)

Supplementary Figure S15 – Forest plot: FDA approved Interventions for cataplexy (cataplexy rate change)

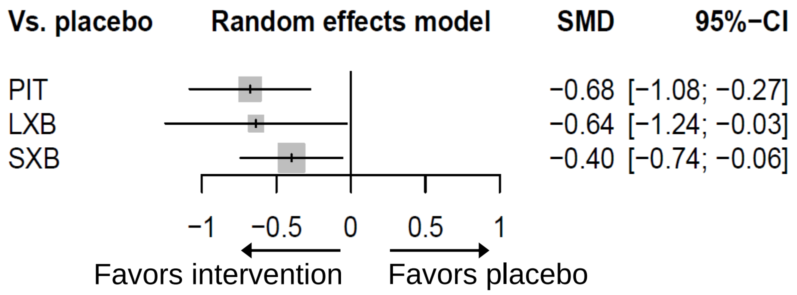

PIT=pitolisant; LXB=lower-sodium oxybate; SXB=sodium oxybate

Supplementary Table S17 – League table: FDA approved Interventions for cataplexy (cataplexy rate change)

**Pitolisant**			
-0.04<br>(-0.77 - 0.69)	**Lower-sodium oxybate**		
-0.28<br>(-0.81 - 0.25)	-0.24<br>(-0.94 - 0.46)	**Sodium oxybate**	
**-0.68<br>(-1.08 - -0.27)**	**-0.64<br>(-1.24 - -0.03)**	**-0.40<br>(-0.74 - -0.06)**	**Placebo**

Interventions were ranked in a descending order of P-scores. Estimates for change in cataplexy rate were shown in standardized mean difference with 95% confidence interval.

Change in cataplexy rate– sensitivity analysis

Supplementary Figure S16 – Forest plot: crossover design excluded (cataplexy rate change)

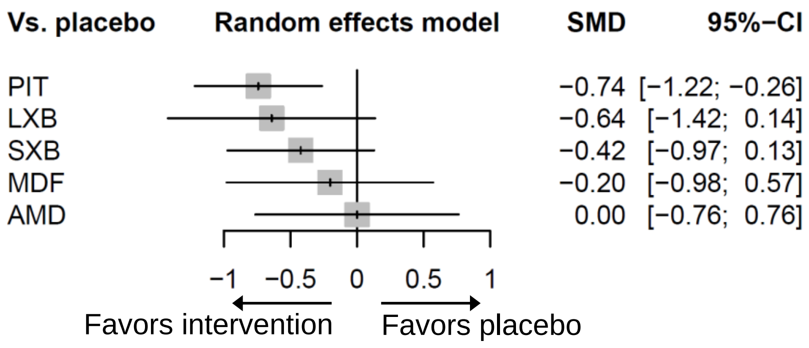

PIT=pitolisant; LXB=lower-sodium oxybate; SXB=sodium oxybate; MDF=modafinil; AMD=armodafinil

Supplementary Table S18 – League table: crossover design excluded (cataplexy rate change)

**Pitolisant**					
-0.10 (-1.01 - 0.81)	**Lower-sodium oxybate**				
-0.32 (-1.05 - 0.41)	-0.22 (-1.17 - 0.74)	**Sodium oxybate**			
-0.54 (-1.31 - 0.24)	-0.44 (-1.53 - 0.66)	-0.22 (-1.17 - 0.73)	**Modafinil**		
-0.74 (-1.64 - 0.16)	-0.64 (-1.73 - 0.45)	-0.42 (-1.36 - 0.52)	-0.20 (-1.29 - 0.88)	**Armodafinil**	
**-0.74 (-1.22 - -0.26)**	-0.64 (-1.42 - 0.14)	-0.42 (-0.97 - 0.13)	-0.20 (-0.98 - 0.57)	0.00 (-0.76 - 0.76)	**Placebo**

Interventions were ranked in a descending order of P-scores. Estimates for change in cataplexy rate were shown in standardized mean difference with 95% confidence interval.

Supplementary Figure S17 – Forest plot: withdrawal design excluded (cataplexy rate change)

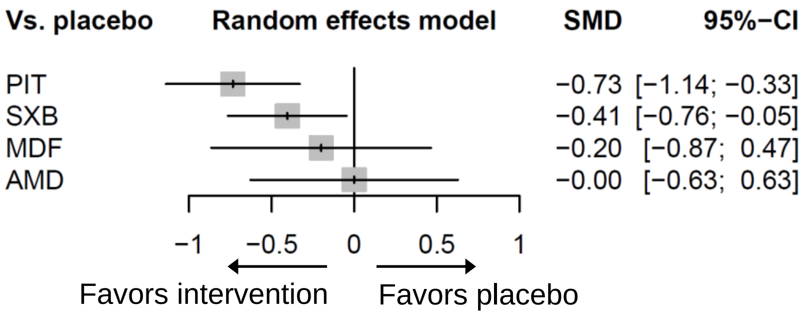

PIT=pitolisant; SXB=sodium oxybate; MDF=modafinil; AMD=armodafinil

Supplementary Table S19 – League table: withdrawal design excluded (cataplexy rate change)

**Pitolisant**				
-0.33<br>(-0.87 - 0.21)	**Sodium oxybate**			
-0.53<br>(-1.20 - 0.13)	-0.21<br>(-0.96 - 0.55)	**Modafinil**		
-0.73<br>(-1.48 - 0.01)	-0.41<br>(-1.13 - 0.32)	-0.20<br>(-1.12 - 0.72)	**Armodafinil**	
**-0.73<br>(-1.14 - -0.33)**	**-0.41<br>(-0.76 - -0.05)**	-0.20<br>(-0.87 - 0.47)	-0.00<br>(-0.63 - 0.63)	**Placebo**

Interventions were ranked in a descending order of P-scores. Estimates for change in cataplexy rate were shown in standardized mean difference with 95% confidence interval.

Clinical Global Impression of Change

Supplementary Figure S18 – Net heat plot: CGI-C

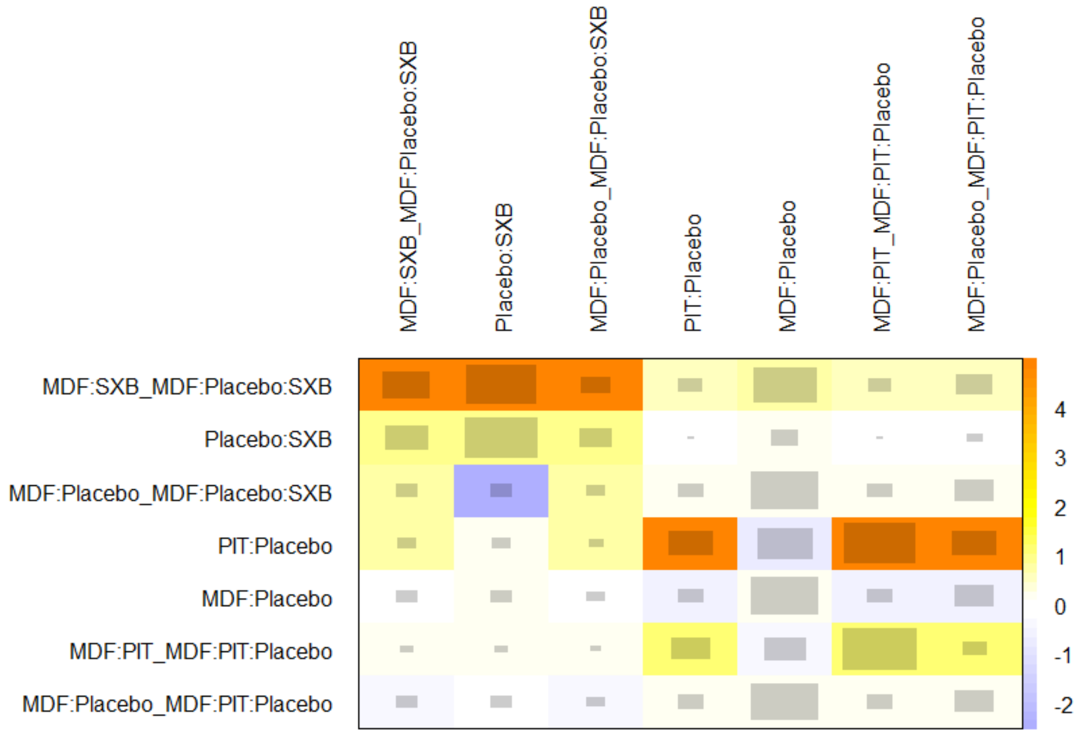

MDF=modafinil; PIT=pitolisant; SXB=sodium oxybate

Graphical display locates potential sources of inconsistency between direct and indirect evidence.

Inconsistency is represented as color, which red being the highest and blue the lowest.
The gray squares' area represents the contribution of the direct estimate of one design in the column to the network estimate in a row.
Pairwise comparisons within the three-arm designs are pointed out by "_".

Supplementary Table S20 – Assessment of inconsistency: CGI-C

Comparison	k	Prop	NMA	Direct	Indir.	Diff	z	p-value
AMD:LXB	0	0	0.57	.	0.57	.	.	.
AMD:MDF	0	0	1.37	.	1.37	.	.	.
AMD:PIT	0	0	1.29	.	1.29	.	.	.
AMD:Placebo	1	1	2.17	2.17	.	.	.	.
AMD:SOF	0	0	1.08	.	1.08	.	.	.
AMD:SXB	0	0	1.04	.	1.04	.	.	.
LXB:MDF	0	0	2.42	.	2.42	.	.	.
LXB:PIT	0	0	2.27	.	2.27	.	.	.
LXB:Placebo	1	1	3.82	3.82	.	.	.	.
LXB:SOF	0	0	1.9	.	1.9	.	.	.
LXB:SXB	0	0	1.83	.	1.83	.	.	.
MDF:PIT	1	0.69	0.94	1.17	0.57	2.07	1.7901	0.0734
MDF:Placebo	4	0.92	1.58	1.54	2.08	0.74	-0.6611	0.5085
MDF:SOF	0	0	0.78	.	0.78	.	.	.
MDF:SXB	1	0.3	0.76	0.4	1	0.4	-2.2278	0.0259
PIT:Placebo	2	0.79	1.69	1.84	1.23	1.49	0.8834	0.377
PIT:SOF	0	0	0.84	.	0.84	.	.	.
PIT:SXB	0	0	0.81	.	0.81	.	.	.
Placebo:SOF	3	1	0.5	0.5	.	.	.	.
Placebo:SXB	3	0.91	0.48	0.53	0.16	3.26	2.1715	0.0299
SOF:SXB	0	0	0.96	.	0.96	.	.	.

AMD=armodafinil; LXB=lower-sodium oxybate; MDF=modafinil; PIT=pitolisant; SOF=solriamfetol; SXB=sodium oxybate

Comparison: Treatment comparison
k: Number of studies providing direct evidence
Prop: Direct evidence proportion
NMA: Estimated treatment effect (RR) in network meta-analysis
Direct: Estimated treatment effect (RR) derived from direct evidence
Indir.: Estimated treatment effect (RR) derived from indirect evidence
Diff: Difference between direct and indirect treatment estimates
z: z-value of test for disagreement (direct versus indirect)
p-value: p-value of test for disagreement (direct versus indirect)

Supplementary Figure S19 – Funnel plot: CGI-C

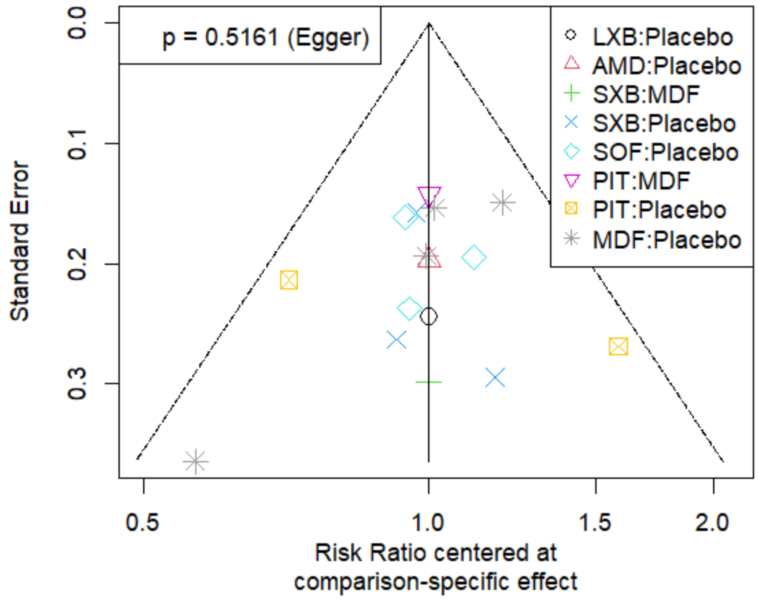

LXB=lower-sodium oxybate; AMD=armodafinil; SXB=sodium oxybate; MDF=modafinil; SOF=solriamfetol; PIT=pitolisant

Clinical Global Impression of Change – subgroup analyses

Supplementary Figure S20 – Network graph: Trials included participants with cataplexy (CGI-C)

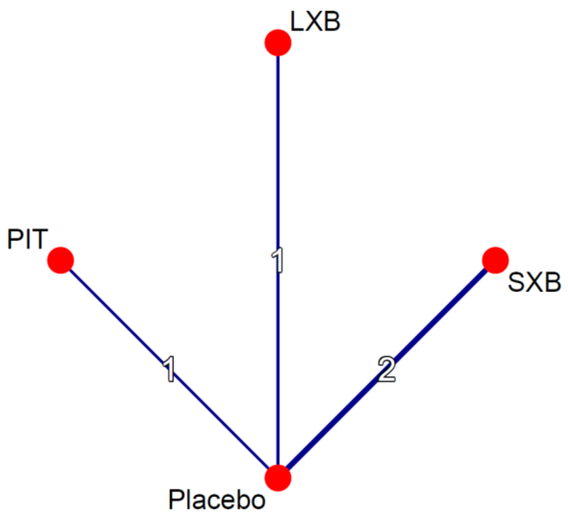

LXB=lower-sodium oxybate; PIT=pitolisant; SXB=sodium oxybate

Nodes: Interventions

Lines Head-to-head comparisons

The width of lines: Number of trials studying the comparison

Supplementary Table S21 – Assessment of inconsistency: Trials included participants with cataplexy (CGI-C)

Comparison	k	Prop	NMA	Direct	Indir.	Diff	z	p-value
PIT:LXB	0	0	0.7616	.	0.7616	.	.	.
Placebo:LXB	1	1	0.2615	0.2615	.	.	.	.
SXB:LXB	0	0	0.4686	.	0.4686	.	.	.
PIT:Placebo	1	1	2.912	2.912	.	.	.	.
PIT:SXB	0	0	1.6253	.	1.6253	.	.	.
Placebo:SXB	2	1	0.5581	0.5581	.	.	.	.

PIT=pitolisant; LXB=lower-sodium oxybate; SXB=sodium oxybate

Comparison: Treatment comparison
k: Number of studies providing direct evidence
Prop: Direct evidence proportion
NMA: Estimated treatment effect (RR) in network meta-analysis
Direct: Estimated treatment effect (RR) derived from direct evidence
Indir.: Estimated treatment effect (RR) derived from indirect evidence
Diff: Difference between direct and indirect treatment estimates
z: z-value of test for disagreement (direct versus indirect)
p-value: p-value of test for disagreement (direct versus indirect)

Supplementary Figure S21 – Forest plot: Trials included participants with cataplexy (CGI-C)

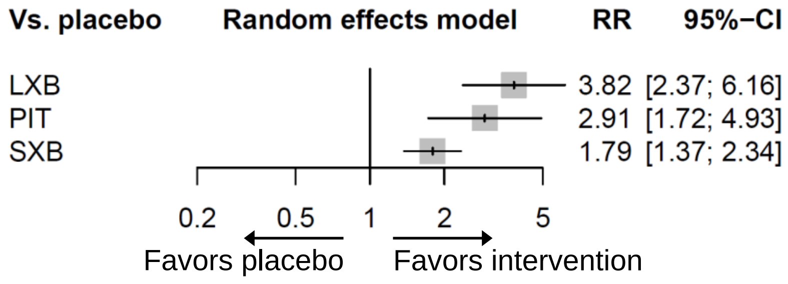

LXB=lower-sodium oxybate; PIT=pitolisant; SXB=sodium oxybate

Supplementary Table S22 – League table: Trials included participants with cataplexy (CGI-C)

**Lower-sodium oxybate**			
1.31 (0.65 - 2.67)	**Pitolisant**		
**2.13 (1.24 - 3.68)**	1.63 (0.90 - 2.93)	**Sodium oxybate**	
**3.82 (2.37 - 6.16)**	**2.91 (1.72 - 4.93)**	**1.79 (1.37 - 2.34)**	**Placebo**

Interventions were ranked in a descending order of P-scores. Estimates for CGI-C were shown in risk ratio with 95% confidence interval.

Clinical Global Impression of Change – sensitivity analyses

Supplementary Figure S22 – Forest plot: crossover design excluded (CGI-C)

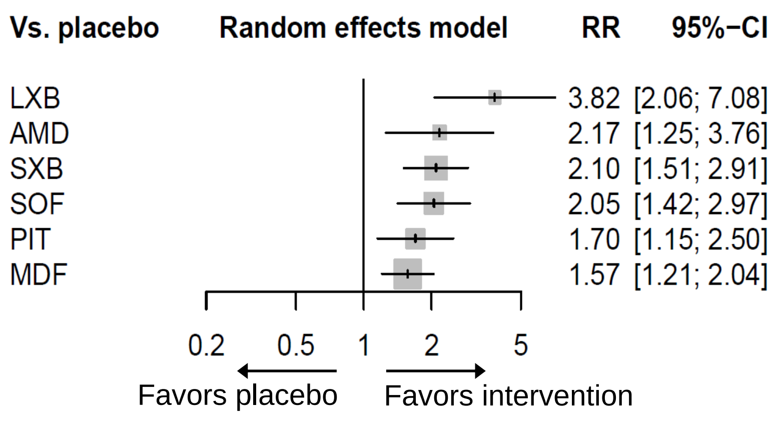

LXB=lower-sodium oxybate; AMD=armodafinil; SXB=sodium oxybate; SOF=solriamfetol; PIT=pitolisant; MDF=modafinil

Supplementary Table S23 – League table: crossover design excluded (CGI-C)

**Lower-sodium oxybate**						
1.76 (0.77 - 4.02)	**Armodafinil**					
1.82 (0.91 - 3.67)	1.04 (0.55 - 1.97)	**Sodium oxybate**				
1.86 (0.91 - 3.83)	1.06 (0.55 - 2.05)	1.02 (0.62 - 1.67)	**Solriamfetol**			
**2.25 (1.09 - 4.66)**	1.28 (0.65 - 2.51)	1.23 (0.75 - 2.03)	1.21 (0.71 - 2.06)	**Pitolisant**		
**2.44 (1.25 - 4.76)**	1.38 (0.75 - 2.55)	1.33 (0.90 - 1.98)	1.31 (0.83 - 2.06)	1.08 (0.73 - 1.60)	**Modafinil**	
**3.82 (2.06 - 7.08)**	**2.17 (1.25 - 3.76)**	**2.10 (1.51 - 2.91)**	**2.05 (1.42 - 2.97)**	**1.70 (1.15 - 2.50)**	**1.57 (1.21 - 2.04)**	**Placebo**

Interventions were ranked in a descending order of P-scores. Estimates for CGI-C were shown in risk ratio with 95% confidence interval.

Supplementary Figure S23– Forest plot: withdrawal design excluded (CGI-C)

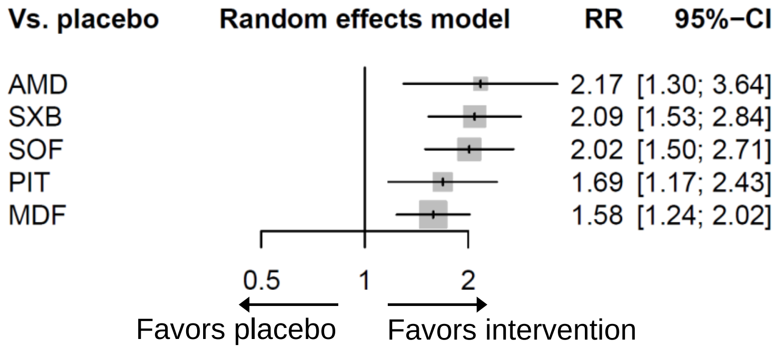

AMD=armodafinil; SXB=sodium oxybate; SOF=solriamfetol; PIT=pitolisant; MDF=modafinil

Supplementary Table S24 – League table: withdrawal design excluded (CGI-C)

**Armodafinil**					
1.04<br>(0.57 - 1.90)	**Sodium oxybate**				
1.08<br>(0.59 - 1.96)	1.04<br>(0.68 - 1.59)	**Solriamfetol**			
1.29<br>(0.68 - 2.42)	1.24<br>(0.78 - 1.98)	1.19<br>(0.75 - 1.91)	**Pitolisant**		
1.37<br>(0.78 - 2.43)	1.32<br>(0.91 - 1.91)	1.27<br>(0.87 - 1.87)	1.07<br>(0.74 - 1.54)	**Modafinil**	
**2.17<br>(1.30 - 3.64)**	**2.09<br>(1.53 - 2.84)**	**2.02<br>(1.50 - 2.71)**	**1.69<br>(1.17 - 2.43)**	**1.58<br>(1.24 - 2.02)**	**Placebo**

Interventions were ranked in a descending order of P-scores. Estimates for CGI-C were shown in risk ratio with 95% confidence interval.

Supplementary Table S25 – Assessment of inconsistency: Cook 2002, Ahmed 2005a, and Gross 2000 excluded (CGI-C)

Comparison	k	Prop	NMA	Direct	Indir.	Diff	z	p-value
AMD:LXB	0	0	0.5683	.	0.5683	.	.	.
AMD:MDF	0	0	1.3142	.	1.3142	.	.	.
AMD:PIT	0	0	1.2466	.	1.2466	.	.	.
AMD:Placebo	1	1	2.1731	2.1731	.	.	.	.
AMD:SOF	0	0	1.0779	.	1.0779	.	.	.
AMD:SXB	0	0	0.7208	.	0.7208	.	.	.
LXB:MDF	0	0	2.3124	.	2.3124	.	.	.
LXB:PIT	0	0	2.1935	.	2.1935	.	.	.
LXB:Placebo	1	1	3.8235	3.8235	.	.	.	.
LXB:SOF	0	0	1.8965	.	1.8965	.	.	.
LXB:SXB	0	0	1.2682	.	1.2682	.	.	.
MDF:PIT	1	0.71	0.9486	1.1729	0.5572	2.1049	1.59	0.112
MDF:Placebo	3	0.93	1.6535	1.5149	5.1691	0.2931	-1.9	0.057
MDF:SOF	0	0	0.8201	.	0.8201	.	.	.
MDF:SXB	1	0.71	0.5484	0.3968	1.2217	0.3248	-1.66	0.0963
PIT:Placebo	2	0.84	1.7431	1.8493	1.2696	1.4566	0.67	0.5041
PIT:SOF	0	0	0.8646	.	0.8646	.	.	.
PIT:SXB	0	0	0.5782	.	0.5782	.	.	.
Placebo:SOF	3	1	0.496	0.496	.	.	.	.
Placebo:SXB	1	0.72	0.3317	0.4545	0.1462	3.109	1.66	0.0963
SOF:SXB	0	0	0.6687	.	0.6687	.	.	.

AMD=armodafinil; LXB=lower-sodium oxybate; MDF=modafinil; PIT=pitolisant; SOF=solriamfetol; SXB=sodium oxybate

Comparison: Treatment comparison

k: Number of studies providing direct evidence

Prop: Direct evidence proportion

NMA: Estimated treatment effect (RR) in network meta-analysis

Direct: Estimated treatment effect (RR) derived from direct evidence

Indir.: Estimated treatment effect (RR) derived from indirect evidence

Diff: Difference between direct and indirect treatment estimates

z: z-value of test for disagreement (direct versus indirect)

p-value: p-value of test for disagreement (direct versus indirect)

Supplementary Figure S24 – Forest plot: Cook 2002, Ahmed 2005a, and Gross 2000 excluded (CGI-C)

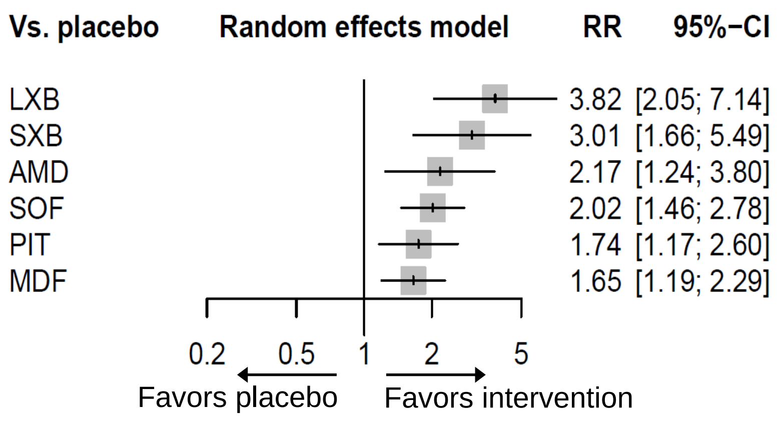

LXB=lower-sodium oxybate; SXB=sodium oxybate; AMD=armodafinil; SOF=solriamfetol; PIT=pitolisant; MDF=modafinil

Supplementary Table S26 – League table: Cook 2002, Ahmed 2005a, and Gross 2000 excluded (CGI-C)

**Lower-sodium oxybate**						
1.27 (0.53 - 3.01)	**Sodium oxybate**					
1.76 (0.76 - 4.07)	1.39 (0.61 - 3.15)	**Armodafinil**				
1.90 (0.94 - 3.83)	1.50 (0.76 - 2.95)	1.08 (0.57 - 2.05)	**Solriamfetol**			
**2.19 (1.04 - 4.61)**	1.73 (0.87 - 3.44)	1.25 (0.63 - 2.48)	1.16 (0.69 - 1.93)	**Pitolisant**		
**2.31 (1.14 - 4.68)**	1.82 (1.00 - 3.32)	1.31 (0.69 - 2.51)	1.22 (0.77 - 1.93)	1.05 (0.70 - 1.60)	**Modafinil**	
**3.82 (2.05 - 7.14)**	**3.01 (1.66 - 5.49)**	**2.17 (1.24 - 3.80)**	**2.02 (1.46 - 2.78)**	**1.74 (1.17 - 2.60)**	**1.65 (1.19 - 2.29)**	**Placebo**

Interventions were ranked in a descending order of P-scores. Estimates for CGI-C were shown in risk ratio with 95% confidence interval.

Adverse events

Supplementary Figure S25 – Forest plot: gastrointestinal adverse events

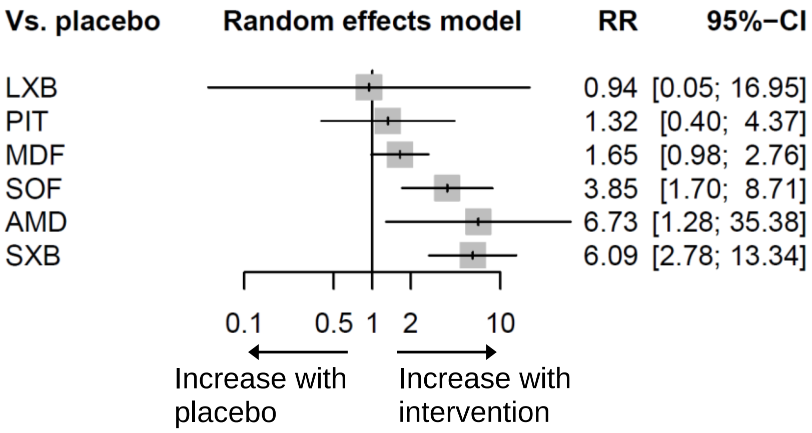

LXB=lower-sodium oxybate; PIT=pitolisant; MDF=modafinil; SOF=solriamfetol; AMD=armodafinil; SXB=sodium oxybate

Supplementary Table S27 – League table: gastrointestinal adverse events

**Placebo**						
1.06 (0.06 - 19.10)	**Lower-sodium oxybate**					
0.76 (0.23 - 2.50)	0.71 (0.03 - 16.26)	**Pitolisant**				
0.61 (0.36 - 1.02)	0.57 (0.03 - 10.78)	0.80 (0.25 - 2.56)	**Modafinil**			
**0.26 (0.11 - 0.59)**	0.24 (0.01 - 4.93)	0.34 (0.08 - 1.46)	0.43 (0.16 - 1.12)	**Solriamfetol**		
**0.15 (0.03 - 0.78)**	0.14 (0.00 - 3.92)	0.20 (0.03 - 1.52)	0.24 (0.04 - 1.39)	0.57 (0.09 - 3.63)	**Armodafinil**	
**0.16 (0.07 - 0.36)**	0.15 (0.01 - 3.09)	**0.22 (0.05 - 0.87)**	**0.27 (0.12 - 0.62)**	0.63 (0.20 - 1.96)	1.10 (0.18 - 6.92)	**Sodium oxybate**

Interventions were ranked in a descending order of P-scores. Estimates for adverse events were shown in risk ratio with 95% confidence interval.

Supplementary Figure S26 – Forest plot: immunologic adverse events

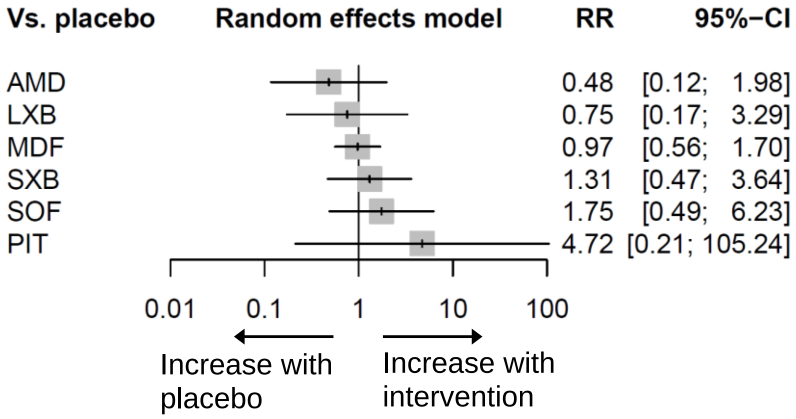

AMD=armodafinil; LXB=lower-sodium oxybate; MDF=modafinil; SXB=sodium oxybate; SOF=solriamfetol; PIT=pitolisant

Supplementary Table S28 – League table: immunologic adverse events

**Armodafinil**						
0.64<br>(0.08 - 4.92)	**Lower-sodium oxybate**					
0.49<br>(0.11 - 2.26)	0.77<br>(0.16 - 3.73)	**Modafinil**				
0.48<br>(0.12 - 1.98)	0.75<br>(0.17 - 3.29)	0.97<br>(0.56 - 1.70)	**Placebo**			
0.37<br>(0.06 - 2.11)	0.58<br>(0.10 - 3.47)	0.75<br>(0.25 - 2.24)	0.77<br>(0.27 - 2.13)	**Sodium oxybate**		
0.27<br>(0.04 - 1.84)	0.43<br>(0.06 - 3.01)	0.56<br>(0.14 - 2.23)	0.57<br>(0.16 - 2.03)	0.75<br>(0.15 - 3.82)	**Solriamfetol**	
0.10<br>(0.00 - 3.08)	0.16<br>(0.01 - 4.95)	0.21<br>(0.01 - 4.83)	0.21<br>(0.01 - 4.71)	0.28<br>(0.01 - 7.27)	0.37<br>(0.01 - 10.59)	**Pitolisant**

Interventions were ranked in a descending order of P-scores. Estimates for adverse events were shown in risk ratio with 95% confidence interval.

Supplementary Figure S27 – Forest plot: musculoskeletal adverse events

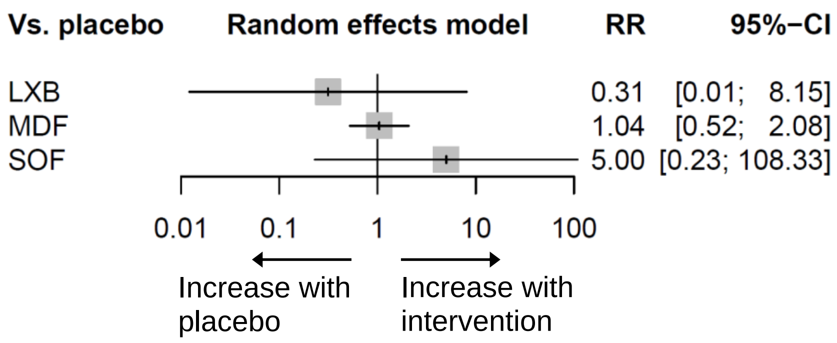

LXB=lower-sodium oxybate; MDF=modafinil; SOF=solriamfetol

Supplementary Table S29 – League table: musculoskeletal adverse events

**Lower-sodium oxybate**			
0.31 (0.01 - 8.15)	**Placebo**		
0.30 (0.01 - 8.41)	0.96 (0.48 - 1.91)	**Modafinil**	
0.06 (0.00 - 5.54)	0.20 (0.01 - 4.33)	0.21 (0.01 - 4.87)	**Solriamfetol**

Interventions were ranked in a descending order of P-scores. Estimates for adverse events were shown in risk ratio with 95% confidence interval.

Supplementary Figure S28 – Forest plot: neurological adverse events

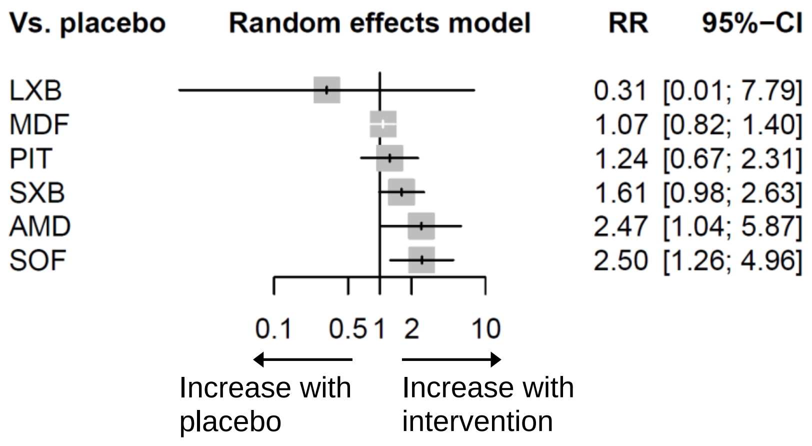

LXB=lower-sodium oxybate; MDF=modafinil; PIT=pitolisant; SXB=sodium oxybate; AMD=armodafinil; SOF=solriamfetol

Supplementary Table S30 – League table: neurological adverse events

**Lower-sodium oxybate**						
0.31 (0.01 - 7.79)	**Placebo**					
0.29 (0.01 - 7.33)	0.93 (0.71 - 1.21)	**Modafinil**				
0.25 (0.01 - 6.67)	0.81 (0.43 - 1.50)	0.87 (0.46 - 1.62)	**Pitolisant**			
0.20 (0.01 - 5.04)	0.62 (0.38 - 1.02)	0.67 (0.39 - 1.14)	0.77 (0.35 - 1.69)	**Sodium oxybate**		
0.13 (0.00 - 3.53)	**0.40 (0.17 - 0.96)**	0.43 (0.18 - 1.07)	0.50 (0.17 - 1.45)	0.65 (0.24 - 1.75)	**Armodafinil**	
0.13 (0.00 - 3.35)	**0.40 (0.20 - 0.79)**	**0.43 (0.21 - 0.90)**	0.50 (0.20 - 1.25)	0.64 (0.28 - 1.49)	0.99 (0.33 - 2.98)	**Solriamfetol**

Interventions were ranked in a descending order of P-scores. Estimates for adverse events were shown in risk ratio with 95% confidence interval.

Supplementary Figure S29 – Forest plot: psychological adverse events

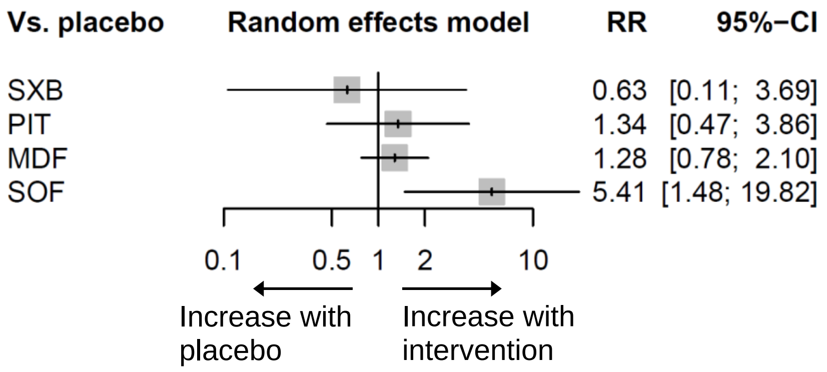

SXB=sodium oxybate; PIT=pitolisant; MDF=modafinil; SOF=solriamfetol

Supplementary Table S31 – League table: psychological adverse events

**Sodium oxybate**				
0.63<br>(0.11 - 3.69)	**Placebo**			
0.47<br>(0.06 - 3.67)	0.75<br>(0.26 - 2.15)	**Pitolisant**		
0.49<br>(0.08 - 3.04)	0.78<br>(0.48 - 1.29)	1.05<br>(0.35 - 3.15)	**Modafinil**	
0.12<br>(0.01 - 1.04)	**0.18**<br>**(0.05 - 0.68)**	0.25<br>(0.05 - 1.32)	**0.24**<br>**(0.06 - 0.95)**	**Solriamfetol**

Interventions were ranked in a descending order of P-scores. Estimates for adverse events were shown in risk ratio with 95% confidence interval.

Supplementary Figure S30 – Forest plot: sleep-related adverse events

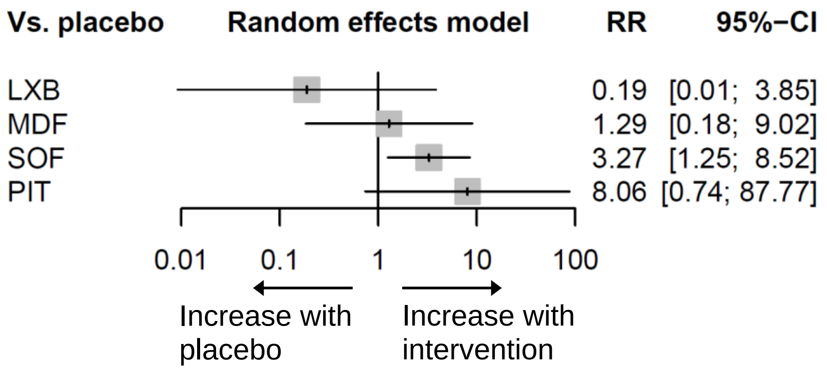

LXB=lower-sodium oxybate; MDF=modafinil; SOF=solriamfetol; PIT=pitolisant

Supplementary Table S32 – League table: sleep-related adverse events

**Lower-sodium oxybate**				
0.19 (0.01 - 3.85)	**Placebo**			
0.15 (0.00 - 5.31)	0.78 (0.11 - 5.45)	**Modafinil**		
0.06 (0.00 - 1.37)	**0.31 (0.12 - 0.80)**	0.39 (0.04 - 3.45)	**Solriamfetol**	
0.02 (0.00 - 1.10)	0.12 (0.01 - 1.35)	0.16 (0.01 - 1.74)	0.41 (0.03 - 5.32)	**Pitolisant**

Interventions were ranked in a descending order of P-scores. Estimates for adverse events were shown in risk ratio with 95% confidence interval.

Supplementary Figure S31 – Forest plot: other adverse events

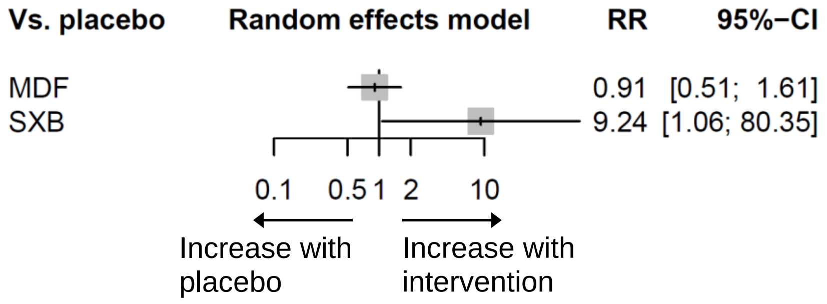

MDF=modafinil; SXB= sodium oxybate

Supplementary Table S33 – League table: other adverse events

**Modafinil**		
0.91<br>(0.51 - 1.61)	**Placebo**	
**0.10**<br>**(0.01 - 0.85)**	0.11<br>(0.01 - 0.94)	**Sodium oxybate**

Interventions were ranked in a descending order of P-scores. Estimates for adverse events were shown in risk ratio with 95% confidence interval.

Supplementary Figure S32 – Forest plot: serious adverse events

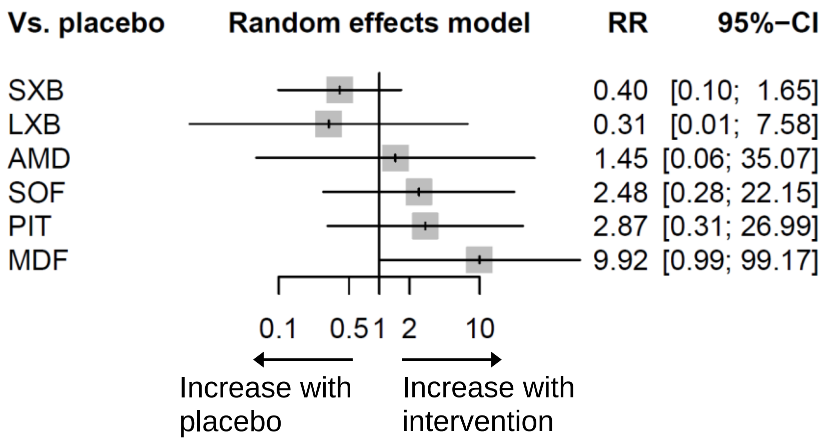

SXB=sodium oxybate; LXB=lower-sodium oxybate; AMD=armodafinil; SOF=solriamfetol; PIT=pitolisant; MDF=modafinil

Supplementary Table S34 – League table: serious adverse events

**Sodium oxybate**						
1.29 (0.04 - 41.78)	**Lower-sodium oxybate**					
0.40 (0.10 - 1.65)	0.31 (0.01 - 7.58)	**Placebo**				
0.28 (0.01 - 9.09)	0.22 (0.00 - 19.59)	0.69 (0.03 - 16.71)	**Armodafinil**			
0.16 (0.01 - 2.20)	0.13 (0.00 - 6.02)	0.40 (0.05 - 3.59)	0.58 (0.01 - 27.83)	**Solriamfetol**		
0.14 (0.01 - 1.99)	0.11 (0.00 - 5.37)	0.35 (0.04 - 3.28)	0.50 (0.01 - 24.84)	0.87 (0.04 - 19.84)	**Pitolisant**	
**0.04 (0.00 - 0.61)**	0.03 (0.00 - 1.61)	0.10 (0.01 - 1.01)	0.15 (0.00 - 7.44)	0.25 (0.01 - 5.99)	0.29 (0.06 - 1.51)	**Modafinil**

Interventions were ranked in a descending order of P-scores. Estimates for adverse events were shown in risk ratio with 95% confidence interval.

Supplementary Figure S33 – Forest plot: trial withdrawal due to AE

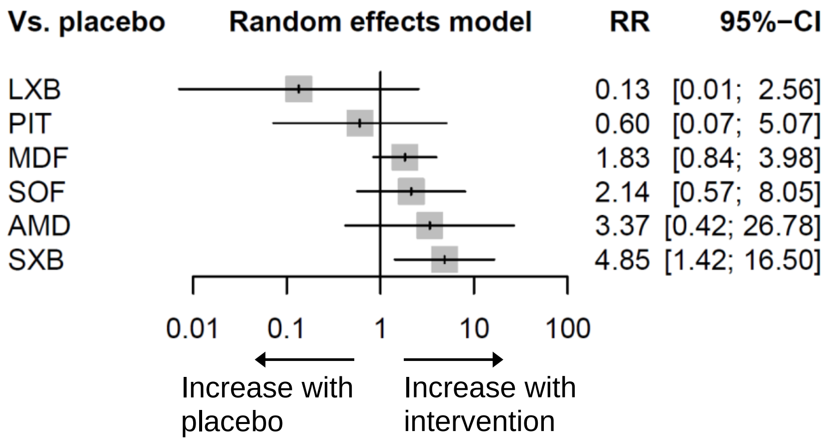

LXB=lower-sodium oxybate; PIT=pitolisant; MDF=modafinil; SOF=solriamfetol; AMD=armodafinil; SXB=sodium oxybate

Supplementary Table S35 – League table: trial withdrawal due to AE

**Lower-sodium oxybate**						
0.22 (0.01 - 8.41)	**Pitolisant**					
0.13 (0.01 - 2.56)	0.60 (0.07 - 5.07)	**Placebo**				
0.07 (0.00 - 1.55)	0.33 (0.04 - 2.91)	0.55 (0.25 - 1.19)	**Modafinil**			
0.06 (0.00 - 1.59)	0.28 (0.02 - 3.47)	0.47 (0.12 - 1.76)	0.86 (0.18 - 3.98)	**Solriamfetol**		
0.04 (0.00 - 1.47)	0.18 (0.01 - 3.50)	0.30 (0.04 - 2.36)	0.54 (0.06 - 4.98)	0.63 (0.05 - 7.44)	**Armodafinil**	
**0.03 (0.00 - 0.67)**	0.12 (0.01 - 1.40)	**0.21 (0.06 - 0.70)**	0.38 (0.11 - 1.33)	0.44 (0.07 - 2.68)	0.69 (0.06 - 7.72)	**Sodium oxybate**

Interventions were ranked in a descending order of P-scores. Estimates for adverse events were shown in risk ratio with 95% confidence interval.

Supplementary Figure S34 – Forest plot: any AE occurred

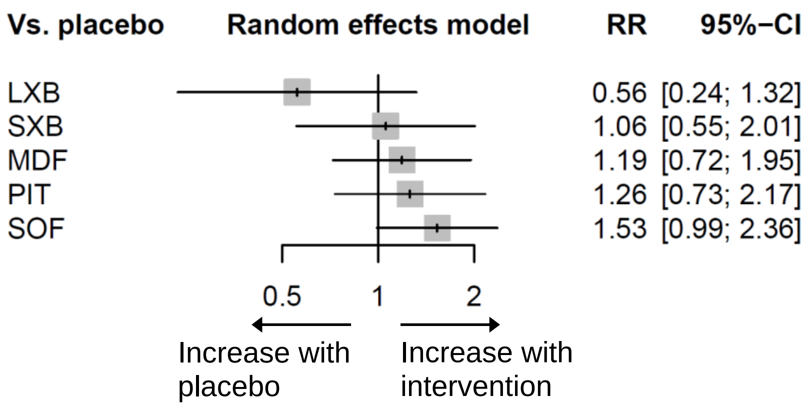

LXB=lower-sodium oxybate; SXB=sodium oxybate; MDF=modafinil; PIT=pitolisant; SOF=solriamfetol

Supplementary Table S36 – League table: any AE occurred

**Lower-sodium oxybate**					
0.56 (0.24 - 1.32)	**Placebo**				
0.53 (0.18 - 1.55)	0.95 (0.50 - 1.80)	**Sodium oxybate**			
0.47 (0.17 - 1.27)	0.84 (0.51 - 1.39)	0.89 (0.46 - 1.71)	**Modafinil**		
0.44 (0.16 - 1.22)	0.79 (0.46 - 1.37)	0.84 (0.38 - 1.83)	0.94 (0.53 - 1.68)	**Pitolisant**	
**0.36 (0.14 - 0.95)**	0.65 (0.42 - 1.01)	0.69 (0.32 - 1.50)	0.78 (0.40 - 1.50)	0.82 (0.41 - 1.65)	**Solriamfetol**

Interventions were ranked in a descending order of P-scores. Estimates for adverse events were shown in risk ratio with 95% confidence interval.

References

1. Bogan, *Efficacy and safety of calcium, magnesium, potassium, and sodium oxybates (lower-sodium oxybate [LXB]; JZP-258) in a placebo-controlled, double-blind, randomized withdrawal study in adults with narcolepsy with cataplexy.* Sleep, 2021. **44**(3).
2. Bogan, *Effect of oral JZP-110 (ADX-N05) treatment on wakefulness and sleepiness in adults with narcolepsy.* Sleep Med, 2015. **16**(9): p. 1102-8.
3. Ruoff, *Effect of Oral JZP-110 (ADX-N05) on Wakefulness and Sleepiness in Adults with Narcolepsy: A Phase 2b Study.* Sleep, 2016. **39**(7): p. 1379-87.
4. Thorpy, *A randomized study of solriamfetol for excessive sleepiness in narcolepsy.* Ann Neurol, 2019. **85**(3): p. 359-370.
5. Dauvilliers, *Pitolisant versus placebo or modafinil in patients with narcolepsy: a double-blind, randomised trial.* Lancet Neurol, 2013. **12**(11): p. 1068-75.
6. Szakacs, *Safety and efficacy of pitolisant on cataplexy in patients with narcolepsy: a randomised, double-blind, placebo-controlled trial.* Lancet Neurol, 2017. **16**(3): p. 200-207.
7. Harsh, *The efficacy and safety of armodafinil as treatment for adults with excessive sleepiness associated with narcolepsy.* Curr Med Res Opin, 2006. **22**(4): p. 761-74.
8. Fry, *Randomized trial of modafinil for the treatment of pathological somnolence in narcolepsy.* Annals of Neurology, 1998. **43**(1): p. 88-97.
9. Gross, *Randomized trial of modafinil as a treatment for the excessive daytime somnolence of narcolepsy: US Modafinil in Narcolepsy Multicenter Study Group.* Neurology, 2000. **54**(5): p. 1166-75.
10. Moldofsky, *A randomized trial of the long-term, continued efficacy and safety of modafinil in narcolepsy.* Sleep Med, 2000. **1**(2): p. 109-116.
11. Saletu, *EEG-tomographic studies with LORETA on vigilance differences between narcolepsy patients and controls and subsequent double-blind, placebo-controlled studies with modafinil.* J Neurol, 2004. **251**(11): p. 1354-63.
12. Ahmed and X.I.S. Group, *Further evidence supporting the use of sodium oxybate for the treatment of cataplexy: a double-blind, placebo-controlled study in 228 patients.* Sleep Med, 2005b. **6**(5): p. 415-21.
13. Black, *Sodium oxybate improves excessive daytime sleepiness in narcolepsy.* Sleep, 2006. **29**(7): p. 939-46.
14. Cook, *A randomized, double blind, placebo-controlled multicenter trial comparing the effects of three doses of orally administered sodium oxybate with placebo for the treatment of narcolepsy.* Sleep, 2002. **25**(1): p. 42-9.
15. Scrima, *Efficacy of gamma-hydroxybutyrate versus placebo in treating narcolepsy-cataplexy: double-blind subjective measures.* Biol Psychiatry, 1989. **26**(4): p. 331-43.

16. Miller, J.L., *Modafinil approved for narcolepsy.* Am J Health Syst Pharm, 1999. **56**(4): p. 304.
17. Black and etal., *A 12-month, open-label, multicenter extension trial of orally administered sodium oxybate for the treatment of narcolepsy.* Sleep, 2003. **26**(1): p. 31-5.
18. Rosenberg, R., M. Baladi, and M. Bron, *Clinically relevant effects of solriamfetol on excessive daytime sleepiness: a posthoc analysis of the magnitude of change in clinical trials in adults with narcolepsy or obstructive sleep apnea.* J Clin Sleep Med, 2021. **17**(4): p. 711-717.
19. Doghramji, K., et al., *Pitolisant in combination with other medications for the management of narcolepsy.* Sleep Medicine, 2019. **64**: p. S94.
20. Sagaspe, P., et al., *Maintenance of Wakefulness Test, real and simulated driving in patients with narcolepsy/hypersomnia.* Sleep Med, 2019. **55**: p. 1-5.
21. Thorpy, *Corrigendum: A Randomized Study of Solriamfetol for Excessive Sleepiness in Narcolepsy.* Ann Neurol, 2020. **87**(1): p. 157.
22. Dauvilliers, Y., et al., *Solriamfetol for the Treatment of Excessive Daytime Sleepiness in Participants with Narcolepsy with and without Cataplexy: Subgroup Analysis of Efficacy and Safety Data by Cataplexy Status in a Randomized Controlled Trial.* CNS Drugs, 2020. **34**(7): p. 773-784.
23. Arnulf, I. and E. Mignot, *Sodium oxybate for excessive daytime sleepiness in narcolepsy-cataplexy.* Sleep, 2004. **27**(7): p. 1242-3.
24. Bauer, E., et al., *The safety and tolerability of pitolisant in the treatment of excessive daytime sleepiness and cataplexy in adult patients with narcolepsy: an open-label, expanded access program in The United States.* Sleep Medicine, 2019. **64**: p. S27.
25. Bauer, E., et al., *Safety and tolerability of pitolisant in the treatment of adult patients with narcolepsy: An open-label, expanded access program in the United States.* Neurology, 2020. **94**(15).
26. Beusterien, K.M., et al., *Health-related quality of life effects of modafinil for treatment of narcolepsy.* Sleep, 1999. **22**(6): p. 757-65.
27. Billiard, M., et al., *Modafinil: a double-blind multicentric study.* Sleep, 1994. **17**(8 Suppl): p. S107-12.
28. Black, J., et al., *Sodium oxybate treatment in patients with narcolepsy stratified by the presence of cataplexy: Retrospective subgroup analysis of a randomized clinical trial.* Sleep, 2015. **38**: p. A267.
29. Black and etal., *Sodium oxybate demonstrates long-term efficacy for the treatment of cataplexy in patients with narcolepsy.* Sleep Med, 2004. **5**(2): p. 119-23.
30. Black, J., et al., *Impact of sodium oxybate, modafinil, and combination treatment on excessive daytime sleepiness in patients who have narcolepsy with or without

cataplexy. Sleep Med, 2016. **24**: p. 57-62.

31. Black, J., et al., *Oral JZP-110 (ADX-N05) for the treatment of excessive daytime sleepiness in adults with narcolepsy: Results of a randomised, double-blind, placebo-controlled trial.* Journal of Sleep Research, 2014. **23**: p. 32-33.
32. Black, J., et al., *Oral JZP-110 phase 2b study for the treatment of excessive sleepiness in adults with narcolepsy: Results of a randomized double-blind, placebo-controlled trial.* Sleep Medicine, 2015. **16**: p. S36.
33. Black, J., et al., *Oral ADX-N05 (JZP-110) for excessive daytime sleepiness (EDS) in narcolepsy: A phase 2b study.* Annals of Neurology, 2014. **76**: p. S130-S131.
34. Black, J.E., et al., *The long-term tolerability and efficacy of armodafinil in patients with excessive sleepiness associated with treated obstructive sleep apnea, shift work disorder, or narcolepsy: an open-label extension study.* J Clin Sleep Med, 2010. **6**(5): p. 458-66.
35. AS, B., H. Y, and M. H, *Dose Sparing Effects of Fluoxetine on Methylphenidate for the Treatment of Sleepiness in Narcolepsy.* Sleep, 2001: p. A313.
36. JA, B., D. SP, and J. M, *Sodium oxybate improves slow wave sleep and daytime sleepiness in narcolepsy.* Sleep, 2006: p. 675.
37. Bogan, R., et al., *Efficacy and safety of Jzp-258 In A Phase 3 Double-Blind, Placebo-Controlled, Randomised-Withdrawal Study In Adults With Narcolepsy With Cataplexy.* Sleep Medicine, 2019. **64**: p. S43.
38. Bogan, R.K., *Sodium oxybate, alone and in combination with modafinil, produces significant improvements in sleep architecture in narcolepsy.* Sleep, 2005.
39. Bogan, R.K., et al., *Efficacy and safety of oral ADX-n05 (JZP-110) for the treatment of excessive daytime sleepiness in adults with narcolepsy.* Annals of Neurology, 2014. **76**: p. S129-S130.
40. Bogan, R.K., et al., *A double-blind, placebo-controlled, randomized, cross-over study of the efficacy and safety of ADX-N05 for the treatment of excessive daytime sleepiness in adult subjects with narcolepsy.* Sleep, 2013. **36**: p. A257.
41. Bogan, R.K., et al., *Time to response with sodium oxybate for the treatment of excessive daytime sleepiness and cataplexy in patients with narcolepsy.* J Clin Sleep Med, 2015. **11**(4): p. 427-32.
42. Broughton, R.J., et al., *Randomized, double-blind, placebo-controlled crossover trial of modafinil in the treatment of excessive daytime sleepiness in narcolepsy.* Neurology, 1997. **49**(2): p. 444-51.
43. Caussé, C., Z. Szakacs, and Y. Dauvilliers, *Efcacy of pitolisant on cataplexy: A double-blind, randomised, placebo-controlled trial in patients with narcolepsy (the harmonyctp trial).* Somnologie, 2017. **21**(2): p. S127.
44. Borgharkar, *A clinical trial to study the effects of two drugs, Armodafinil and*

Modafinil in patients with excessive daytime sleepiness associated with narcolepsy. 2010.
45. Dauvilliers, Y., *Novel therapeutic approach in narcolepsy: Clinical trials of an antagonist/inverse agonist of the histamine H3 receptor.* Journal of Sleep Research, 2014. **23**: p. 75.
46. Dauvilliers, Y., et al., *Long-term evaluation of safety and efficacy of pitolisant in narcolepsy: harmony 3 study.* Sleep Medicine, 2019. **64**: p. S85.
47. Dauvilliers, Y., et al., *Long term use of pitolisant to treat narcolepsy: HARMONY III study.* Journal of Sleep Research, 2016. **25**: p. 275.
48. Dauvilliers, Y., et al., *Long-term use of pitolisant to treat patients with narcolepsy: Harmony III Study.* Sleep, 2019. **42**(11).
49. Dauvilliers, Y., et al., *Long-term evaluation of safety and efficacy of pitolisant in narcolepsy: Harmony III study.* Sleep, 2018. **41**: p. A231-A232.
50. Dauvilliers, Y., et al., *Long-term evaluation of safety and efficacy of pitolisant (wakix®), an histamine H3R antagonist, in narcolepsy.* Sleep Medicine, 2017. **40**: p. e75.
51. Dauvilliers, Y., et al., *Long-term evaluation of safety and efficacy of pitolisant in narcolepsy: HARMONY 3 study.* Neurology, 2019. **92**(15).
52. Dauvilliers, Y., et al., *Cataplexy-free days in a phase 3, placebocontrolled, double-blind, randomized withdrawal study of JZP-258 in adults with narcolepsy with cataplexy.* Sleep, 2020. **43**(SUPPL 1): p. A286.
53. Dauvilliers, Y., et al., *Modafinil reduces symptoms of excessive sleepiness in children and adolescents with obstructive sleep apnea or narcolepsy following 6 months of open-label therapy.* Sleep, 2007.
54. Dauvilliers, Y., et al., *Effect of sodium oxybate (SXB), modafinil and combination on disrupted nighttime sleep in narcolepsy.* Sleep Medicine, 2013. **14**: p. e105.
55. Dauvilliers, Y., et al., *Solriamfetol (JZP-110) for treatment of excessive sleepiness in narcoleptic patients with and without cataplexy: Results from a randomized, phase 3, clinical trial.* Sleep, 2018. **41**: p. A229-A230.
56. Dauvilliers, Y., et al., *Changes in cataplexy frequency by prior therapy in a phase 3, double-blind, placebo-controlled, randomised withdrawal study of JZP-258 in adults with narcolepsy with cataplexy.* Sleep Medicine, 2019. **64**: p. S86.
57. Dauvilliers, Y., et al., *Efficacy of pitolisant on cataplexy: A double blind, randomised, placebo controlled trial in patients with narcolepsy (the HARMONY-CTP trial).* Journal of Sleep Research, 2016. **25**: p. 255.
58. Davis, C.W., et al., *Efficacy of pitolisant in patients with high burden of narcolepsy symptoms: pooled analysis of short-term, placebo-controlled studies.* Sleep Med, 2021. **81**: p. 210-217.
59. Duntley, S.P. and M.J. Morrissey, *Evaluation of the safety of sodium oxybate oral*

solution versus placebo in patients with narcolepsy. Neurology, 2005.
60. Emsellem, H., et al., *Measures of function, work productivity, and quality of life from a phase 3 study of solriamfetol (JZP-110) in patients with narcolepsy.* Sleep, 2018. **41**: p. A230-A231.
61. Emsellem, H., et al., *Quality of life, functional evaluation, and work productivity in patients with narcolepsy: results from a phase 3 study of solriamfetol (JZP-110).* Journal of Sleep Research, 2018. **27**: p. 183.
62. Emsellem, H.A. and U.S.M.S. Grp, *Efficacy and safety profiles of modafinil maintained during long-term (40 and 88 weeks) treatment of excessive daytime sleepiness associated with narcolepsy.* Neurology, 2000.
63. Bassetti. *Prospective, randomized, double-blind study, parallel-group, multi-center trial assessing the effects of escalating doses of BF2.649 and BF2.649 add on Modafinil on cataplexy in patients with narcolepsy (HARMONY II) - Harmony II.* 2009; Available from: https://trialsearch.who.int/?TrialID=EUCTR2008-007845-29-DE.
64. Avadel. *A study to test whether Sodium Oxybate for Extended Release Oral Suspension (FT218) is safe and treats Excessive Daytime Sleepiness and Catalexy in subjects with Narcolepsy*. 2016; Available from: http://www.who.int/trialsearch/Trial2.aspx?TrialID=EUCTR2016-000359-29-DE.
65. Euctr, F.I., *Study of the Efficacy and Safety of Xyrem in Pediatric Subjects with Narcolepsy with Cataplexy.* https://trialsearch.who.int/Trial2.aspx?TrialID=EUCTR2014-001389-93-FI, 2014.
66. Euctr, N.L., *Study to evaluate the efficacy in reducing residual Excessive Daytime Sleepiness (EDS) and the number of cataplectic episodes (for patients with cataplexy) of BF2.649 (pitolisant) in narcoleptic children from 6 to less than 18 years.* https://trialsearch.who.int/Trial2.aspx?TrialID=EUCTR2013-001506-29-NL, 2016.
67. Feldman, N., *Sodium oxybate, alone and in combination with modafinil, is safe and well-tolerated for the treatment of narcolepsy.* Sleep, 2005.
68. Feldman, N.T., *Sodium oxybate therapy significantly improves the excessive daytime sleepiness associated with narcolepsy.* Sleep, 2001.
69. Foldvary-Schaefer, N., et al., *Jzp-258 dose titration and transition from sodium oxybate in a placebo-controlled, double-blind, randomized withdrawal study in adult participants with narcolepsy with cataplexy.* Annals of Neurology, 2020. **88**(SUPPL 25): p. S251.
70. Foldvary-Schaefer, N., et al., *Quality of life in phase 3, placebo-controlled, double-blind, randomized withdrawal study of JZP-258 in adults with narcolepsy with cataplexy.* Sleep, 2020. **43**(SUPPL 1): p. A281-A282.
71. Foldvary-Schaefer, N., et al., *Long-term effects of solriamfetol on functioning and work productivity in participants with excessive daytime sleepiness associated with*

narcolepsy. Neurology, 2020. **94**(15).
72. Fry, J.M., *A new alternative in the pharmacological management of somnolence: A phase III study of modafinil in narcolepsy.* Annals of Neurology, 1996.
73. J. Harsh, R.R.a.G.E.N., *A 12 week randomized double blind placebo controlled study of armodafinil in adults with excessive sleepiness associated with narcolepsy.* Sleep medicine, 2005.
74. R. Hayduk, J.H.a.T.R., *Armodafinil improves subjective measures of sleepiness in patients with excessive sleepiness associated with obstructive sleep apnea/hypopnea syndrome, narcolepsy, and shift work sleep disorder.* Sleep, 2006.
75. Mitler, R.H.a.M., *Sodium oxybate therapy improves the quality of life of narcolepsy patients.* Sleep, 2001.
76. Hidalgo, H., et al., *Pitolisant as therapeutic option for narcolepsy: Results from the German compassionate-program 2015/16.* Journal of Sleep Research, 2016. **25**: p. 366.
77. Harsh, M.H.a.J., *Modafinil improves steer clear performance in narcolepsy.* Sleep, 2004.
78. M. Hirshkowitz, J.S., B. Corser and P. Sahota, *Long-term (136 weeks) safety and efficacy of modafinil for the treatment of excessive daytime sleepiness associated with narcolepsy.* Sleep, 2001.
79. Hong, S.C., et al., *Clinical and polysomnographic features in DQB1*0602 positive and negative narcolepsy patients: results from the modafinil clinical trial.* Sleep Med, 2000. **1**(1): p. 33-39.
80. S. Hull, T.R.a.T.R., *Armodafinil does not affect intended sleep as determined by polysomnography in patients with excessive sleepiness.* Sleep, 2006.
81. Ivanenko, A. and L. Kek, *Treatment of excessive daytime sleepiness with modafinil and armodafinil in early-onset narcolepsy comorbid with psychiatric disorders.* Journal of the American Academy of Child and Adolescent Psychiatry, 2017. **56**(10): p. S219.
82. Joo, E.Y., et al., *Effect of modafinil on cerebral blood flow in narcolepsy patients.* Sleep, 2008. **31**(6): p. 868-73.
83. Kovacević-Ristanović, R. and T.J. Kuźniar, *Use of sodium oxybate (Xyrem in patients with dual diagnosis of narcolepsy and sleep apnea.* Sleep Med, 2010. **11**(1): p. 5-6.
84. Laffont, F., G. Mayer, and M. Minz, *Modafinil in diurnal sleepiness. A study of 123 patients.* Sleep, 1994. **17**(8 Suppl): p. S113-5.
85. Lammers, G.J., et al., *Sodium oxybate is an effective and safe treatment for narcolepsy.* Sleep Med, 2010. **11**(1): p. 105-6; author reply 106-8.
86. Lavault, S., et al., *Benefit and risk of modafinil in idiopathic hypersomnia vs. narcolepsy with cataplexy.* Sleep Med, 2011. **12**(6): p. 550-6.

87. Malhotra, A., et al., *Long-term effects of solriamfetol on quality of life in participants with excessive daytime sleepiness associated with narcolepsy or obstructive sleep apnoea.* Journal of Sleep Research, 2020. **29**(SUPPL 1).
88. Malhotra, A., et al., *A long-term safety and maintenance of efficacy study of solriamfetol (JZP-110) in the treatment of excessive sleepiness in subjects with narcolepsy or obstructive sleep apnea.* Sleep, 2018. **41**: p. A230.
89. Malhotra, A., et al., *Weight change associated with solriamfetol treatment of excessive daytime sleepiness in participants with narcolepsy or obstructive sleep apnea.* Sleep, 2019. **42**: p. A246.
90. Malhotra, A., et al., *Long-term effects of solriamfetol on functioning and work productivity in participants with excessive daytime sleepiness associated with narcolepsy.* Annals of Neurology, 2020. **88**(SUPPL 25): p. S253.
91. Mamelak, M., et al., *A 12-week open-label, multicenter study evaluating the safety and patient-reported efficacy of sodium oxybate in patients with narcolepsy and cataplexy.* Sleep Med, 2015. **16**(1): p. 52-8.
92. Mamelak, M., et al., *A 12-week, open-label study evaluating sodium oxybate (SXB) in patients with narcolepsy.* Annals of Neurology, 2014. **76**: p. S131-S132.
93. Mayer, G., et al., *The effect of sodium oxybate on the motor activity in sleep in patients with narcolepsy.* Sleep, 2010. **33**: p. A273.
94. Mayer, G., et al., *Muscle activity during wake and sleep in narcolepsy patients treated with sodium oxybate.* Sleep, 2011. **34**: p. A4.
95. Meskill, G.J., *0755 Pitolisant (Wakix) is an Effective Anti-Cataplexy Agent in Narcolepsy Type 1.* Sleep, 2020. **43**(Supplement_1): p. A287-A287.
96. Mitler, M.M., et al., *Treatment of narcolepsy: objective studies on methylphenidate, pemoline, and protriptyline.* Sleep, 1986. **9**(1 Pt 2): p. 260-4.
97. Montplaisir, J., et al., *Effects of sodium oxybate on measures of daytime sleepiness in narcolepsy patients: Preliminary evidence of dose-related improvements.* Sleep, 2001.
98. Lee, Y.-J., *Provigil (Modafinil) Study by Taiwan Biotech Co*. 2005.
99. Corporation, A., *A Safety and Effectiveness Study of a Single Dose of JNJ-17216498 in Patients With Narcolepsy*. 2007.
100. Dauvilliers, Y., *Safety and Efficacy of THN102 on Sleepiness in Narcoleptic Patients*. 2016.
101. Ramaekers, J., *Study Assessing Effects of JZP-110 on Driving Performance in the Treatment of Excessive Sleepiness in Narcolepsy*. 2016.
102. Trotti, L.M., *Modafinil Versus Amphetamines for the Treatment of Narcolepsy Type 2 and Idiopathic Hypersomnia*. 2018.
103. Nicollet, A., et al., *Treatment of narcolepsy with modafinil: A long-term open study in 244 patients.* Neurology, 2000.

104. Parkes, J.D. and G.W. Fenton, *Levo(-) amphetamine and dextro(+) amphetamine in the treatment of narcolepsy.* J Neurol Neurosurg Psychiatry, 1973. **36**(6): p. 1076-81.
105. Pepin, J.L., et al., *Long-term efficacy of solriamfetol for excessive sleepiness in narcolepsy or obstructive sleep apnea.* European Respiratory Journal, 2019. **54**.
106. Pepin, J.L., et al., *A long-term study of the safety and maintenance of efficacy of solriamfetol (JZP-110) for treatment of excessive sleepiness associated with narcolepsy or obstructive sleep apnoea.* Journal of Sleep Research, 2018. **27**: p. 183-184.
107. Philip, P., et al., *Modafinil improves real driving performance in patients with hypersomnia: a randomized double-blind placebo-controlled crossover clinical trial.* Sleep, 2014. **37**(3): p. 483-7.
108. Richter, R.W., *A clinical trial of modafinil in patients with narcolepsy: Safety and improvement in illness.* Psychopharmacology Bulletin, 1997.
109. Rosenberg, R., M. Baladi, and M. Bron, *Clinically relevant effects of solriamfetol on excessive daytime sleepiness: A posthoc analysis of the magnitude of change in clinical trials in adults with narcolepsy or obstructive sleep apnea.* Journal of Clinical Sleep Medicine, 2021. **17**(4): p. 711-717.
110. Rosenberg, R., M. Baladi, and M. Bron, *Clinically relevant effects of solriamfetol on excessive daytime sleepiness: A post-hoc analysis of the magnitude of change in a clinical trial of adults with narcolepsy.* Annals of Neurology, 2020. **88**(SUPPL 25): p. S256.
111. Rosenberg, R., M. Baladi, and M. Bron, *Clinically relevant effects of solriamfetol on excessive daytime sleepiness: Post hoc analyses of clinical trials in narcolepsy or obstructive sleep apnoea.* Journal of Sleep Research, 2020. **29**(SUPPL 1).
112. Rosenberg, R., et al., *Clinically relevant effects of solriamfetol on excessive sleepiness: A post hoc responder analysis of clinical trials in adults with narcolepsy or obstructive sleep apnea.* Journal of Managed Care and Specialty Pharmacy, 2018. **24**(10 A): p. S53.
113. Rosenberg, R., et al., *Armodafinil sustains wakefulness throughout the day in patients with excessive sleepiness associated with narcolepsy.* Neurology, 2009.
114. Rosenberg, R., et al., *Incidence and duration of common adverse events in 2 solriamfetol phase 3 studies for treatment of excessive daytime sleepiness in obstructive sleep apnoea and narcolepsy.* Sleep Medicine, 2019. **64**: p. S326.
115. Rosenberg, R. and J. Walsleben, *Armodafinil reduces daytime fatigue associated with excessive sleepiness in patients with obstructive sleep apnea/hypopnea syndrome or narcolepsy.* Sleep, 2006.
116. Roth, T., et al., *Effect of sodium oxybate on sleep stage shifts and sleep quality in patients with narcolepsy.* Sleep Medicine, 2013. **14**: p. e249.

117. Roth, T., et al., *Armodafinil improves wakefulness throughout the day in patients with excessive sleepiness associated with narcolepsy.* Sleep, 2009. **32**: p. A245.
118. Roth, T., et al., *Armodafinil sustains wakefulness throughout the day in patients with excessive sleepiness associated with narcolepsy.* Annals of Neurology, 2008: p. S29-S29.
119. Roy, A., et al., *Time course of improvement in excessive daytime sleepiness and cataplexy during treatment with pitolisant in patients with narcolepsy.* Sleep, 2020. **43**(SUPPL 1): p. A291-A292.
120. Ruoff, C., et al., *JZP-110 significantly improves 20-minute-censored maintenance of wakefulness test sleep latency in narcolepsy patients.* Journal of Sleep Research, 2016. **25**: p. 178.
121. Ruoff, C., et al., *JZP-110 has a large effect size on the maintenance of wakefulness test in patients with narcolepsy independent of 20-or 40-minute censorship of the data.* Sleep, 2016. **39**: p. A241.
122. Ruoff, C., et al., *Evaluation of the effect of JZP-110 in patients with narcolepsy assessed using the Maintenance of Wakefulness Test censored to 20 minutes.* Sleep Med, 2017. **35**: p. 12-16.
123. Sagaspe, P., et al., *How real road-driving performance, simulated driving performance, and maintenance of wakefulness test are related in narcolepsy/hypersomnia patients?* Sleep Medicine, 2017. **40**: p. e287.
124. Sagaspe, P., et al., *Maintenance of Wakefulness Test, real and simulated driving in narcolepsy/hypersomnia patients.* Journal of Sleep Research, 2018. **27**: p. 9.
125. Sahota, P., *Modafinil for the long-term treatment of excessive daytime sleepiness in narcolepsy.* Annals of Neurology, 1997.
126. Saletu, M., et al., *Low-resolution brain electromagnetic tomography (LORETA) identifies brain regions linked to psychometric performance under modafinil in narcolepsy.* Psychiatry Res, 2007. **154**(1): p. 69-84.
127. Scharf, M.B., *Assessment of sodium oxybate for the long-term treatment of narcolepsy.* Sleep, 2001.
128. Schwartz, J.R., M. Hirshkowitz, and W. Schmidt-Nowara, *Modafinil Dosing During Long-Term Treatment of Excessive Sleepiness in Narcolepsy and Obstructive Sleep Apnea.* CHEST, 2004. **126**(4): p. 903S.
129. Schwartz, J.R., M.T. Nelson, and R.J. Hughes, *Modafinil improves executive function in narcolepsy patients with excessive sleepiness.* Sleep, 2003.
130. Schwartz, J.R., et al., *Modafinil improves cognition in narcolepsy patients with excessive sleepiness*, in *156th annual meeting of the american psychiatric association*. 2003: San francisco CA.
131. Schweitzer, P., et al., *Solriamfetol (JZP-110) in the treatment of excessive sleepiness in*

narcolepsy and obstructive sleep apnoea: maintenance of wakefulness test results across the day. Journal of Sleep Research, 2018. **27**: p. 179-180.
132. Scrima, L., et al., *Patient and clinician global impressions of change in disease status were correlated in a clinical trial of JZP-110 treatment for narcolepsy.* Annals of Neurology, 2015. **78**: p. S116.
133. Shapiro, C., et al., *A long-term safety and maintenance of efficacy study of solriamfetol (JZP-110) for the treatment of excessive sleepiness associated with narcolepsy or obstructive sleep apnea.* Annals of Neurology, 2018. **84**: p. S232-S233.
134. Strollo, P.J., et al., *Effects of solriamfetol on 24-hour blood pressure patterns in participants with excessive daytime sleepiness associated with narcolepsy.* Sleep, 2020. **43**(SUPPL 1): p. A293.
135. Stultz, D.J., et al., *A one year observational early access pitolisant study of excessive daytime sleepiness in narcolepsy.* Sleep, 2020. **43**(SUPPL 1): p. A287.
136. Szakacs, Z., et al., *Pitolisant efficacy on cataplexy: A double blind, randomised, placebo controlled trial in patients with narcolepsy (the harmony-CTP trial).* Sleep Medicine, 2017. **40**: p. e322.
137. Takahashi, Y., *A Double-blind, Cross-over Study of the Effects of Ritalin, Sp-732 and Placebo on Narcolepsy.* Rinsho hyoka (clinical evaluation), 1979.
138. Thorpy, M., *Orally administered sodium oxybate for the treatment of narcolepsy.* Curr Neurol Neurosci Rep, 2004. **4**(2): p. 155-6.
139. Thorpy, M., et al., *A randomized, placebo-controlled, phase 3 study of solriamfetol (JZP-110) in patients with narcolepsy: Effects on wakefulness, excessive sleepiness, and health-related quality-of-life.* Neuropsychopharmacology, 2017. **43**: p. S254-S255.
140. Thorpy, M.J., et al., *A randomized, placebo-controlled, phase 3 study of the safety and efficacy of solriamfetol (JZP-110) for the treatment of excessive sleepiness in patients with narcolepsy.* Sleep Medicine, 2017. **40**: p. e327.
141. Thorpy, M.J., et al., *Safety and efficacy of JZP-110 for treatment of excessive sleepiness in narcolepsy: Results of a randomized, placebo-controlled, phase 3 study.* Annals of Neurology, 2017. **82**: p. S113.
142. Thorpy, M.J., et al., *Pooled analyses from 12-week randomised, controlled studies of solriamfetol in the treatment of excessive daytime sleepiness in participants with OSA or narcolepsy.* Sleep Medicine, 2019. **64**: p. S385-S386.
143. Thorpy, M.J., et al., *Changes in cataplexy frequency by therapy at study entry in a phase 3, placebo-controlled, double-blind, randomized withdrawal study of JZP-258 in adults with narcolepsy with cataplexy.* Neurology, 2020. **94**(15).
144. Thorpy, M.J., et al., *A Randomized, placebo-controlled, phase 3 study of the safety and efficacy of solriamfetol (JZP-110) for the treatment of excessive sleepiness (ES) in participants with narcolepsy types 1 and 2 (NT1/2).* Neurology, 2018. **90**(15).

145. van der Heide, A., et al., *Comparing Treatment Effect Measurements in Narcolepsy: The Sustained Attention to Response Task, Epworth Sleepiness Scale and Maintenance of Wakefulness Test.* Sleep, 2015. **38**(7): p. 1051-8.
146. Villa, K., et al., *Use of the 36-item short-form health survey in a clinical trial: Evaluation of quality-of-life in patients with narcolepsy with cataplexy treated with sodium oxybate.* Journal of Managed Care and Specialty Pharmacy, 2015. **21**: p. S43-S44.
147. Vinckenbosch, F., et al., *Effects of solriamfetol on driving performance in participants with narcolepsy.* Sleep, 2020. **43**(SUPPL 1): p. A290.
148. Weaver, T.E., *Sodium oxybate therapy for narcolepsy improves patient quality of life.* Sleep, 2005.
149. Weaver, T.E. and N. Cuellar, *A randomized trial evaluating the effectiveness of sodium oxybate therapy on quality of life in narcolepsy.* Sleep, 2006. **29**(9): p. 1189-94.
150. Weaver, T.E., et al., *Relationship between efficacy endpoints and measures of functional status and health-related quality of life (HRQoL) in narcolepsy patients treated for excessive sleepiness.* Journal of Sleep Research, 2018. **27**: p. 298-299.
151. Wesnes, K., G. Niebler, and S. Arora, *The cognitive effects of armodafinil in patients with excessive sleepiness associated with osahs, narcolepsy, and shift work sleep disorder.* European Neuropsychopharmacology, 2005.
152. Wesnes, K., G. Niebler, and S. Arora, *The cognitive effects of armodafinil in patients with excessive sleepiness associated with obstructive sleep apnea/hypopnea, narcolepsy, and shift work sleep disorder.* Journal of Psychopharmacology, 2005.
153. Winter, W., et al., *Cardiac safety profile of pitolisant in patients with narcolepsy.* Sleep, 2020. **43**(SUPPL 1): p. A283.
154. Yan, B., J.T. Guo, and L.P. Li, *Clinical effect of venlafaxine combined with methylphenidate hydrochloride on narcolepsy.* Chinese Journal of Contemporary Neurology and Neurosurgery, 2013. **13**(11): p. 961-963.
155. Scrima, L., et al., *The effects of gamma-hydroxybutyrate on the sleep of narcolepsy patients: a double-blind study.* Sleep, 1990. **13**(6): p. 479-90.
156. Mitler, M.M., R. Hajdukovic, and M.K. Erman, *Treatment of narcolepsy with methamphetamine.* Sleep, 1993. **16**(4): p. 306-17.
157. Black, *The nightly administration of sodium oxybate results in significant reduction in the nocturnal sleep disruption of patients with narcolepsy.* Sleep Med, 2009. **10**(8): p. 829-35.
158. Black, *The nightly use of sodium oxybate is associated with a reduction in nocturnal sleep disruption: a double-blind, placebo-controlled study in patients with narcolepsy.* J Clin Sleep Med, 2010. **6**(6): p. 596-602.
159. Schwartz, *Effects of modafinil on wakefulness and executive function in patients with*

narcolepsy experiencing late-day sleepiness. Clin Neuropharmacol, 2004. **27**(2): p. 74-9.

160. Schwartz, J.R., et al., *Dosing regimen effects of modafinil for improving daytime wakefulness in patients with narcolepsy.* Clin Neuropharmacol, 2003. **26**(5): p. 252-7.

161. Dauvilliers, Y., *Patient Narcoleptic Treated With BF2.649 (Pitolisant) in add-on to Sodium Oxybate (HARMONY IV)*. 2013.

162. Emsellem, *Measures of functional outcomes, work productivity, and quality of life from a randomized, phase 3 study of solriamfetol in participants with narcolepsy.* Sleep Med, 2020. **67**: p. 128-136.

163. Saletu, *EEG-mapping differences between narcolepsy patients and controls and subsequent double-blind, placebo-controlled studies with modafinil.* Eur Arch Psychiatry Clin Neurosci, 2005. **255**(1): p. 20-32.
